# Substantially Altered Local and Systemic Immunity in Ischemia‐Free Versus Conventional Liver Transplantation

**DOI:** 10.1002/advs.202502854

**Published:** 2026-01-27

**Authors:** Tao Luo, Shuai He, Shirui Chen, Dongmei Ye, Shibo Zhang, Di Liu, Dawei Zou, Linhe Wang, Huadi Chen, Jinghong Xu, Huanjie Liu, Ruiting Liu, Xi Tan, Yitong Wang, Jiayi Zeng, Runbing Mo, Yuexin Li, Yuyi Zhang, Tanyiyang Ruan, Tielong Wang, Zehua Jia, Yu Jia, Tongdi Fang, Yimou Lin, Xiaorui Liu, Yunhua Tang, Maogen Chen, Qiang Zhao, Anbin Hu, Weiqiang Ju, Yi Ma, Dongping Wang, Xiaofeng Zhu, Schlegel Andrea, Yuan Zhai, Kwan Man, Tullius G. Stefan, Yifang Gao, Jinxin Bei, Xiaoshun He, Zhiyong Guo

**Affiliations:** ^1^ Organ Transplant Center The First Affiliated Hospital Sun Yat‐sen University Guangzhou China; ^2^ Guangdong Provincial Key Laboratory of Organ Medicine Guangzhou China; ^3^ Guangdong Provincial International Cooperation Base of Science and Technology (Organ Transplantation) Guangzhou China; ^4^ NHC Key Laboratory of Assisted Circulation Sun Yat‐sen University Guangzhou China; ^5^ State Key Laboratory of Oncology in South China Guangdong Provincial Clinical Research Center for Cancer Sun Yat‐sen University Cancer Center Guangzhou China; ^6^ Immunobiology & Transplant Science Center Department of Surgery Houston Methodist Hospital Houston Methodist Research Institute Houston USA; ^7^ Transplantation Center Digestive Disease and Surgery Institute, and Department of Immunology Cleveland Clinic Lerner Research Institute Cleveland USA; ^8^ Transplant Surgery College of Medicine Medical University of South Carolina Charleston South Carolina USA; ^9^ Department of Surgery HKU‐Shenzhen Hospital & Department of Surgery LKS Faculty of Medicine The University of Hong Kong Hong Kong China; ^10^ Division of Transplant Surgery Department of Surgery Brigham and Women's Hospital Harvard Medical School Boston Massachusetts USA

**Keywords:** allograft rejection, ischemia‐free liver transplantation, ischemia‐reperfusion injury, macrophages, monocytes, neutrophils, single‐cell sequencing

## Abstract

Ischemia‐free liver transplant (IFLT) has been developed to reduce ischemia‐reperfusion injury (IRI). This study aims to investigate how this procedure impacts local and systemic immunity compared to conventional liver transplantation (CLT). Immunohistochemistry, immunofluorescence staining, single‐cell RNA sequencing (scRNA‐seq), and multiplex cytokine are used to illustrate distinct local and systemic immunity. In contrast to CLT, IFLT reduces neutrophil infiltration and neutrophil extracellular trap formation in grafts. By constructing an immune cell chimerism atlas, we reveal that IFLT reduces recipient‐derived monocyte infiltration by suppressing ANXA1‐FPR1 signaling through the STAT3‐HIF‐1α pathway, thereby attenuating inflammatory responses in graft monocytes. Additionally, IFLT confers graft protection by upregulating *HMOX1* expression in monocytes and macrophages. Peripherally, IFLT significantly reduces the expression of MHC II molecules in circulating monocytes. Accordingly, CD8^+^ effector T cell composition, T helper 1 (Th1) and Th17 cytokine levels are reduced, while regulatory T cell (Treg) composition and Th2 cytokine levels are increased in IFLT versus CLT recipients. These results show that IFLT profoundly affects local and systemic immunity in liver transplantation. Recipient‐circulating monocytes might play a key role in the interaction between graft IRI and allograft rejection.

## Graphical abstract

This study investigates how ischemia‐free liver transplantation (IFLT) shapes both local and systemic immune responses compared with conventional liver transplantation (CLT). Our findings demonstrate that IFLT confers significant immunological protection by attenuating the STAT3‐HIF‐1α axis and enhancing the Heme‐HMOX1 pathway, two regulatory networks that link improved graft‐level protection with coordinated systemic immune modulation.

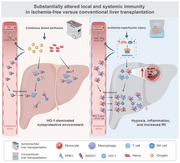

## 1 Introduction

Organ transplantation is the standard treatment for patients with organ failure [1]. However, graft ischemia‐reperfusion injury (IRI) remains an inevitable challenge in conventional organ transplantation [2]. IRI significantly contributes to post‐transplant early graft dysfunction or non‐function, thus compromising transplant outcomes and limiting organ utilization [3]. Moreover, graft IRI is considered a major trigger of allograft rejection [4, 5]. Therefore, understanding the mechanisms of IRI and developing effective therapies are of critical clinical importance in organ transplantation.

The mechanism of graft IRI in transplantation has been characterized by the significant involvement of various innate and adaptive immune cells at different stages [6]. Studies have shown that both donor‐ and recipient‐derived immune cells play key roles in the pathogenesis of IRI [7, 8]. Graft IRI not only impacts local but also systemic immunity, contributing to remote injuries [9]. Recent advances in single‐cell RNA sequencing (scRNA‐seq) technologies have provided important insights into the dynamic immune responses following liver transplantation (LT), including IRI complications [10, 11], transplant rejection [12, 13, 14], and biliary strictures [15]. However, the impact of early IRI on both donor‐ and recipient‐derived cells, and how these cellular responses collectively shape transplant immunity, remains poorly understood.

Based on normothermic machine perfusion (NMP) and surgical innovation, our group introduced the concept of ischemia‐free organ transplantation (IFOT) in the liver [16], kidney [17], and heart [18]. The results of our recent randomized controlled trial have shown that graft IRI‐related complications are reduced in ischemia‐free liver transplantation (IFLT) compared to conventional liver transplantation (CLT) [19]. Detailed laboratory analyses have demonstrated that graft IRI is largely mitigated by IFLT [20]. However, how the ischemia‐free procedure impacts the local and systemic immunity during graft IRI is largely unknown.

In this study, through comprehensive single‐cell sequencing analysis of liver grafts and peripheral bloods from IFLT and CLT recipients, we documented the profound impact of the ischemia‐free procedure on local and systemic immunity.

## 2 Materials and Methods

### 2.1 Sample Collection and Comprehensive Analyses

Donor liver biopsies and blood samples were collected from patients who received IFLT and CLT in our first non‐randomized and randomized clinical trial [19, 21] (Table S1–S4). Details of the IFLT and CLT procedures were provided in our published randomized trials [19]. During IFLT, the liver was procured after an in situ NMP circuit was established, preserved using ex situ NMP, and subsequently implanted under in situ NMP. During CLT, the livers were procured after a fast cold flush, preserved at 0–4°C in University of Wisconsin (UW) solution, and implanted under hypothermic and hypoxic conditions.

**FIGURE 1:**
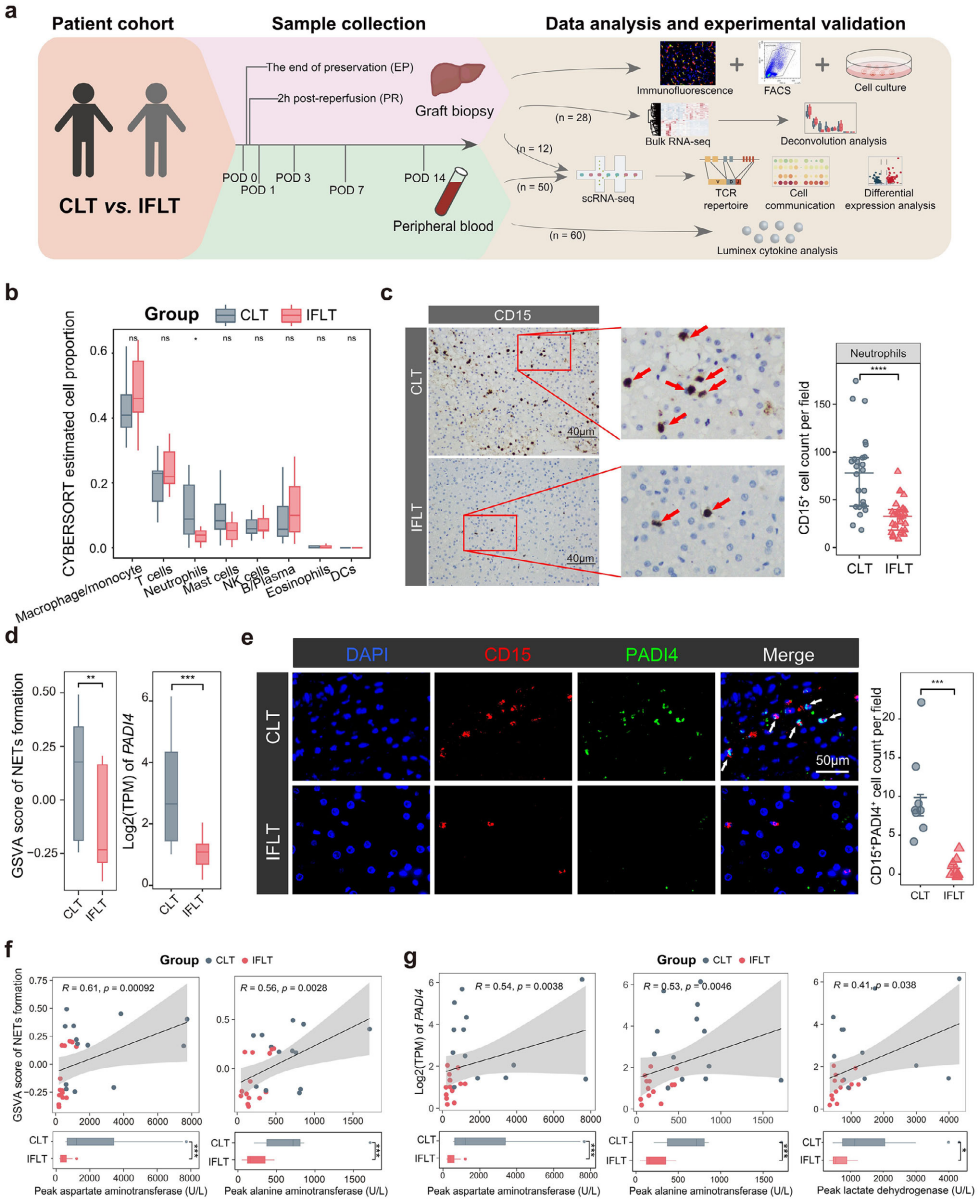
Analysis of intra‐graft immunity in IFLT vs CLT. (a) An experimental schematic diagram of the overall study design. (b) Boxplot shows the proportion of major immune cell clusters estimated by CIBERSORT in IFLT (*n* = 14) and CLT (*n* = 14) grafts. (c) Immunochemistry assay shows neutrophil (CD15^+^) infiltration across CLT (*n* = 27) and IFLT (*n* = 27) grafts. (d) Boxplot shows the GSVA score of NETs formation and the normalized expression of *PADI4* expression in samples across CLT and IFLT grafts. (e) Multi‐color immunofluorescence assay in CLT (*n* = 8) and IFLT (*n* = 8) grafts. (f) The scatter plot shows the Pearson correlation between the GSVA score for NETs formation in grafts and liver enzyme levels in peripheral blood post‐transplantation. A statistic bar plot shows the liver enzyme levels in the peripheral blood post‐transplantation between IFLT (*n* = 14) and CLT (*n* = 14). (g) The scatter plot shows the Pearson correlation between the GSVA score for *PADI4* expression levels and liver enzyme levels in peripheral blood post‐transplantation. Ns, no significance; ^*^ *p* < 0.05; ^**^ *p* < 0.01; ^***^ *p* < 0.001; ^****^ *p* < 0.0001.

For scRNA‐seq, three liver biopsies were collected at the end of preservation (EP) and at 2 h post‐reperfusion (PR) in both IFLT and CLT groups. Additional liver biopsies at PR were obtained from both groups for bulk RNA sequencing (RNA‐seq), immunohistochemistry (IHC), and immunofluorescence (IF) staining. Blood samples were collected at PR and on post‐operative days (POD) 0, 1, 3, 7, and 14 for scRNA‐seq, multiplex cytokine/chemokine analyses, or flow cytometry assays (Figure 1a).

### 2.2 Immunostaining Assays

Human liver tissues were washed with 1x DPBS, fixed in 4% paraformaldehyde (pH 7.0), and embedded in paraffin. Paraffin‐embedded blocks were then cut into 5‐µm thick slides using a pathology slicer (RM2016, Leica Microsystems, Germany) and adhered to glass slides. The glass sections were then deparaffinized, rehydrated, and subjected to a blockade of endogenous peroxidase activity with 3% H_2_O_2_, followed by high‐temperature antigen retrieval. The sections were blocked with 5% bovine serum albumin (9048‐46‐8; Guangzhou Ruishu Biotechnology) at room temperature for 1 h, followed by incubation with primary antibodies overnight at 4°C. Further compensation and analysis were performed using the FlowJo software. Further details regarding the IHC analysis and IF assays can be found in the Supporting Information.

### 2.3 Single‐Cell Suspension Preparation and scRNA‐seq Data Processing

All samples were processed within 1.5 h following our previously established protocols for single‐cell suspension preparation [22]. The standard procedure of the Chromium Single Cell V(D)J Kit V2 was used for single‐cell library preparation. Based on the human reference (genome and VDJ dataset), CellRanger (v5.0.0, 10X Genomics) was used to generate a gene expression matrix for further analysis. Quality control, integration of the dataset, clustering, and downstream analysis were performed using Seurat (v3.2.3) [23]. Please refer to the Supporting Information for the complete protocol of the downstream analysis.

### 2.4 Identification of Cells from Donors and Recipients

After liver transplantation, immune cells from recipients infiltrate donor livers via the blood circulation. To investigate the composition of different immune cells from either the donor or recipient in the grafts in the PR phase, we used the “Souporcell” algorithm to identify cell identities [24]. A full description for “Souporcell” analysis can be found in the Supporting Information.

### 2.5 Multiplex Cytokine/Chemokine Analysis

To separate serum, after collecting blood in tubes containing EDTA anticoagulant, samples were centrifuged immediately at 1,500 × g for 10 min at 4°C. After centrifugation, serum was immediately transferred into sterile polypropylene tubes and stored at −80°C. Serum cytokine concentrations were measured using Procarta Plex (Invitrogen Systems, Carlsbad, CA, USA). The following markers were analyzed: cytokines from T helper cellsTh1/Th2 cells (GM‐CSF, IFN‐γ, IL‐2, IL‐4, IL‐6, LIF, IL‐12p70, IL‐13, IL‐18, and TNF‐α), Th9/Th17/Th22/Treg cells (IL‐9, IL‐10, IL‐17A, IL‐21, IL‐22, and IL‐27), inflammatory cytokines (IFN‐α, IL‐1α, IL‐1β, IL‐1RA, IL‐7, IL‐15, and IL‐31), chemokine (CCL11, CXCL1, CXCL10, CCL3, CCL4, CCL5, and SDF‐1), and growth factors (BDNF, EGF, FGF‐2, NGF‐β, PDGF‐BB, PlGF‐1, SCF, and VEGF‐A). Quantitative cytokine data were obtained according to the manufacturer's instructions (FLEXMAP 3D; Luminex) and evaluated using the xPONENT software (Luminex Corporation, Austin, TX, USA).

### 2.6 Statistical Analysis

Statistical tests were performed using the R software (v4.1.3). The unpaired two‐sided Wilcoxon rank‐sum tests or unpaired two‐sided *t*‐tests were used to estimate the statistical differences between the IFLT and CLT groups. One‐way ANOVA was applied to evaluate differences in Median Fluorescence Intensity (MFI) across three experimental conditions in flow‐cytometry assays. Pearson's correlation coefficient was used to measure the correlation between two different indices. *p* < 0.05 was considered statistically significant.

## 3 Results

### 3.1 Reduced Neutrophil Infiltration and Neutrophil Extracellular Traps Formation in IFLT versus CLT Grafts

To investigate how CLT and IFLT affect the early infiltration of various immune cells in grafts, we estimated the immune cell composition in liver grafts using the “CIBERSORT” algorithm [25] on our previous bulk RNA‐seq data from CLT and IFLT grafts collected at the PR phase [20]. We observed a significantly reduced neutrophil proportion and neutrophil signature score in IFLT compared to CLT grafts (Figure 1b; Figure S1a). Particularly, neutrophil signature genes were preferentially expressed in CLT grafts (Figure S1b). Moreover, IF assays of liver biopsies confirmed a significant decrease in CD15^+^ neutrophil infiltration in IFLT compared to CLT grafts, while no significant difference was observed in CD3^+^ T cells, CD68^+^ macrophages, and CD57^+^ NK cells (Figure 1c; Figure S1c–e).

In human liver transplantation, higher serum levels of the neutrophil extracellular traps (NETs)‐related marker citrullinated histone H3 (H3Cit) are associated with severe graft injury [26]. Gene set variation analysis (GSVA) showed that NETs formation scores were decreased in IFLT versus CLT grafts (Figure 1d). The expression of peptidyl arginine deiminase 4 (*PADI4*), a marker of NETs formation [27], was also significantly lower in IFLT than in CLT grafts (Figure 1d). IF staining confirmed the decreased number of CD15^+^PADI4^+^ neutrophils in IFLT compared to CLT grafts (Figure 1e). Moreover, both NETs formation and *PADI4* expression were positively correlated with post‐transplant peak liver enzyme levels in peripheral blood (Figure 1f,g; Figure S1f). Collectively, at 2 h post‐reperfusion, we documented significantly enhanced neutrophil infiltration and NETs formation in CLT IFLT grafts, which might represent the first wave of inflammation after transplantation.

### 3.2 Chimerism of Donor‐ and Recipient‐Derived Immune Cells in the Grafts Early After IFLT and CLT

**FIGURE 2:**
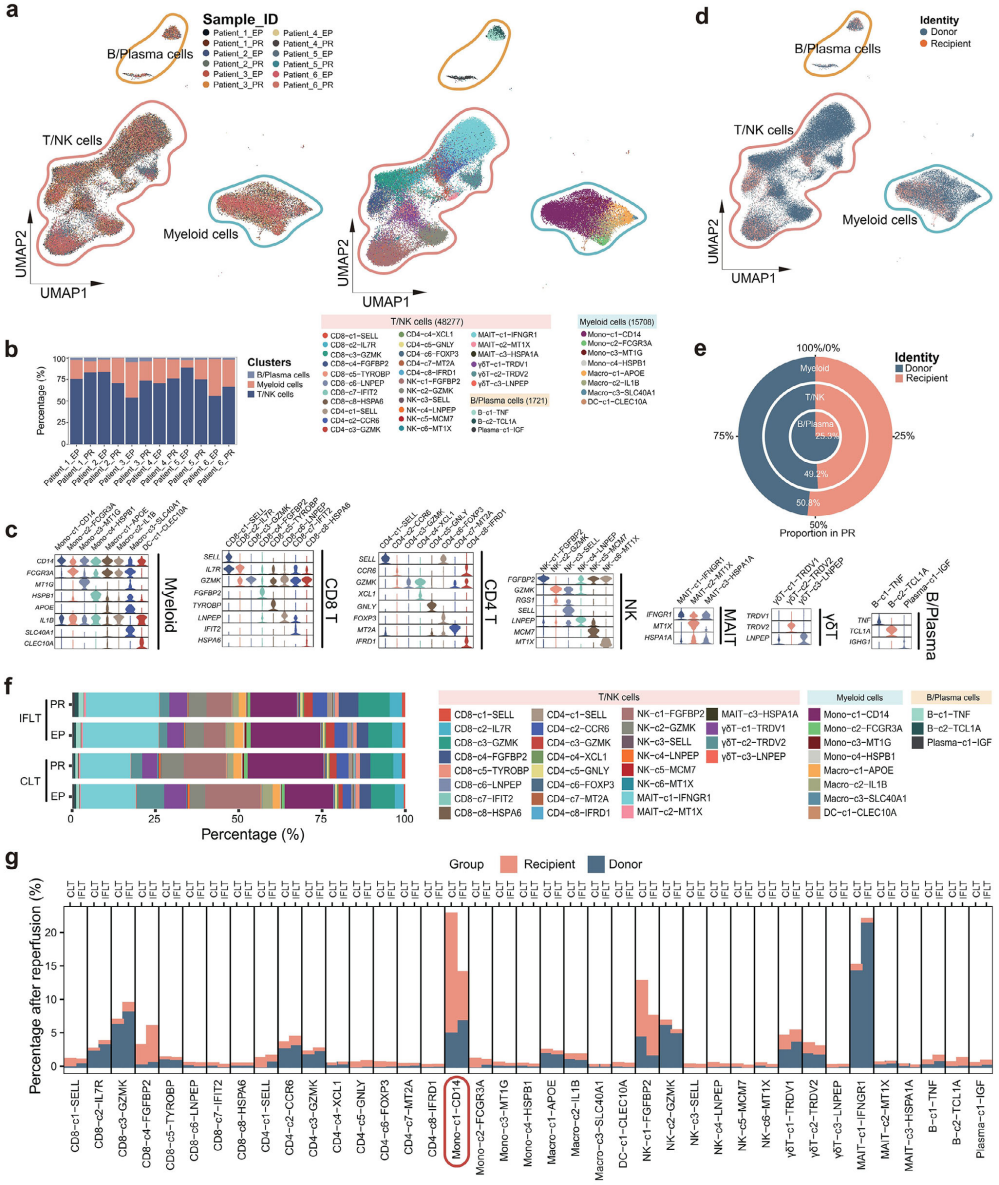
Single‐cell transcriptome map of donor‐ and recipient‐derived immune cells in CLT and IFLT grafts. (a) UMAP plots show single cells, colored by samples (left panel) and cell clusters (right panel). (b) Bar plots show the proportion of major cell clusters in each sample. (c) Violin plots show normalized expression levels of marker genes in each cluster. (d) UMAP plots show major cell clusters, colored by cell origin from donors or recipients. (e) A circular pie chart shows the proportion of cells from donors and recipients at the PR phase. (f) Bar plots show the proportion of 39 subclusters at EP and PR from CLT and IFLT patients. (g) Bar plots show the percentage of donor/recipient‐derived immune cell clusters at the PR phase.

To gain intricate insights into the graft immune microenvironment from a single‐cell perspective, we conducted scRNA‐seq analysis of liver biopsies. Our analysis encompassed the single‐cell transcriptome and TCR sequencing of CD45^+^ cells, leading to a comprehensive dataset containing 65,706 high‐quality single‐cell transcriptomes (Figure 2a,b). Using unsupervised clustering, we identified 39 distinct subtypes delineated by canonical marker genes, including 28 T/NK cell subsets, three B cell subsets, and eight myeloid cell subsets (Figure 2c; Figure S2a). Neutrophils are terminally differentiated and short‐lived immune cells with low RNA contents and high levels of RNases [28, 29], thus limiting the collection of sufficient neutrophils for traditional droplet‐based scRNA‐seq analysis.

Subsequently, we applied the Souporcell pipeline and TCR clone type information to segment the immune cells from all PR liver samples into donor‐ and recipient‐derived clusters (Figure S2b–f). Approximately half of T/NK cells and myeloid cells, and 25.3% of B/plasma cells originated from recipients (Figure 2d,e). The recipient‐derived peripheral immune cells gradually replaced donor‐derived cells, particularly CD8‐c4, Mono‐c1, and NK‐c1 at PR (Figure 2f,g). However, tissue‐resident immune cells, including mucosal‐associated invariant T (MAIT) and NK‐c2 cells, were not replaced by the recipient counterparts (Figure 2g). These data represent the first single‐cell landscape of the early chimera of donor‐ and recipient‐derived immune cells in human liver transplantation.

### 3.3 IFLT Reduces the Hypoxic Status of both Donor‐Derived and Recipient‐Derived Cells through STAT3‐HIF‐1α Signaling Pathway

**FIGURE 3:**
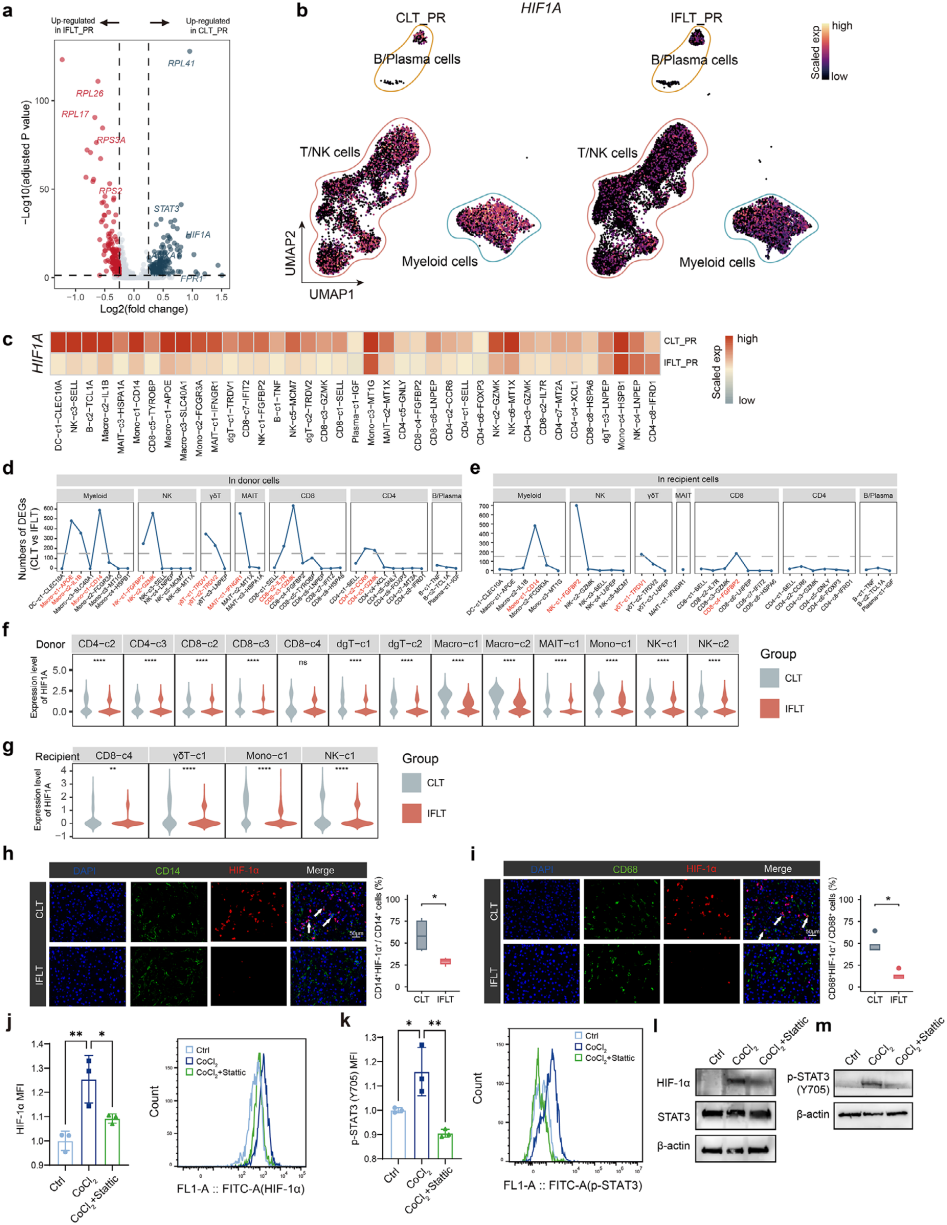
Downregultion of *HIF1A* expression in donor‐ and recipient‐derived immune cells in IFLT grafts. (a) Volcano plot shows DEGs between CLT_PR and IFLT_PR groups in all immune cells. (b) UMAP plots show the expression level of *HIF1A* across CLT_PR and IFLT_PR groups in all immune cells. (c) Heatmap shows normalized *HIF1A* expression across CLT_PR and IFLT_PR groups in all immune cells (d), (e). Line plot shows the numbers of DEGs in donor‐ (d) and recipient‐derived (e) subclusters. (f), (g) Violin plots show *HIF1A* expression levels in donor‐ (f) and recipient‐derived (g) IRIs‐icell clusters between two groups. (h), (i) Multi‐color immunofluorescence staining for CD14 (green) (h), CD68 (green) (i), and HIF‐1α (red) in PR samples across CLT and IFLT patients (CD14: *n* = 5 in CLT, *n* = 3 in IFLT; CD68: *n* = 4 in CLT, *n* = 5 in IFLT). (j), (k) Bar plots showing the expression of HIF‐1α (j) and p‐STAT3 (k) in monocytes under control conditions, CoCl_2_ treatment, or CoCl_2_ plus the STAT3 inhibitor Stattic, measured by flow cytometry (*n* = 3 per group). Representative histogram plots are shown to the right of the scatter plot. (l), (m) Representative Western blot showing HIF‐1α (l), STAT3 (l), and p‐STAT3 (m) expression in monocytes under the same conditions as in j‐k. Monocytes in panels j‐l were isolated from the peripheral blood of healthy donors. β‐actin was used as a loading control. ^*^ *p* < 0.05; ^**^ *p* < 0.01; ^****^ *p* < 0.0001.

To comprehensively characterize the impact of two liver transplantation strategies on graft transcriptomes, we performed differential expression gene (DEGs) analysis comparing CLT with IFLT at PR. We observed that *HIF1A*, a master regulator of cellular homeostatic adaptation to hypoxia [30], was uniformly downregulated across diverse immune cell populations in the IFLT group at 2 h post‐reperfusion (Figure 3a–c). This widespread downregulation suggests that IFLT markedly alleviates hypoxia‐induced stress within the graft.

We identified 12 donor‐derived and 4 recipient‐derived IRI‐sensitive immune cell clusters (IRIs‐icells) based on their substantial numbers of DEGs, which accounted for 89.6% of the intra‐graft donor‐derived immune cells and 69.8% of the intra‐graft recipient‐derived immune cells (Figure 3d,e; Figure S3a,b). These IRIs‐icells shared common DEGs patterns (Figure S3c,d). Gene ontology (GO) analysis revealed distinct biological functions across IRIs‐icell clusters (Figure S3e–j), while also highlighting shared transcriptional responses to ischemic stress. Donor‐derived immune cells in the CLT group exhibited coordinated enrichment of hypoxia‐related pathways (“response to hypoxia,” “response to oxygen levels”), as well as “leukocyte cell–cell adhesion” and “I‐κB kinase/NF‐κB signaling” (Figure S3k,l). Consistent with these findings, oxidative stress– and heat shock–related genes (*NFE2L2*, *HSPA5*, *HSP90AA1*, and *HSP90AB1*), hypoxia‐associated genes (*HIF1A*, *STAT3*, *UCP2*, and *NAMPT*), adhesion molecules (*CD44*, *LGALS1*, and *LGALS3*), and inflammatory regulators (*NFKB1* and *NFKB2*) were upregulated in the CLT group at the PR timepoint (Figure S4a–e). Cell–type–specific alterations were also evident. Notably, the “cell killing” pathway was significantly enriched in the CD8‐c3‐GZMK and NK‐c1‐FGFBP2 clusters in CLT, accompanied by elevated expression of cytotoxic effector molecules (*GZMB*, *PRF1*, *LYST*, *GNLY*, and *GZMM*), consistent with enhanced cytotoxic activity in these subsets (Figures S3k,l, and S4a,b). Importantly, scores for “response to hypoxia” and “cell killing” pathways showed positive correlations with peak liver enzyme levels, indicating that stronger activation of these pathways is associated with more severe hepatic injury (Figure S4f–j).

We observed that *HIF1A* expression was significantly higher in both donor‐ and recipient‐derived cells in CLT compared with IFLT at PR (Figure 3f–g), demonstrating that IFLT markedly alleviates the hypoxia‐driven microenvironment associated with ischemia‐reperfusion. IF staining further confirmed a widespread reduction of HIF‐1α levels across multiple cell types, most prominently in monocytes and macrophages, in IFLT grafts (Figure 3h,i; Figure S5a,b). STRING analysis of shared DEGs comparing PR to ER in both CLT and IFLT groups revealed putative interactions between STAT3 and HIF1A (Figure S5c). Previous studies reported that STAT3 enhances HIF‐1α protein stability under hypoxia [31], and that HIF‐1α accumulates and translocates to the nucleus under low oxygen conditions [32]. Moreover, STAT3 activity and expression were significantly upregulated in donor‐ and recipient‐derived IRIs‐icells in CLT at PR (Figure 3a; Figure S5d,e). To experimentally test whether STAT3 stabilizes HIF‐1α under hypoxia, we treated PBMC‐derived monocytes with Cobaltous chloride (CoCl_2_) to mimic hypoxic conditions. CoCl_2_ induced strong upregulation of HIF‐1α and p‐STAT3 without altering total STAT3 levels (Figure 3j–m; Figure S5f). Importantly, STAT3 inhibition significantly reduced HIF‐1α and p‐STAT3 levels while leaving total STAT3 unchanged (Figure 3j–m; Figure S5f). Consistent with this, higher HIF‐1α and p‐STAT3 levels were observed in CLT monocytes from PBMC compared with IFLT at PR, whereas total STAT3 remained comparable between groups (Figure S5g–k).

Together, these results suggest that STAT3 contributes to HIF‐1α stabilization under hypoxic conditions, and that suppression of the STAT3‐HIF‐1α axis in IFLT may play a key role in reducing IRI‐associated hypoxic stress and downstream inflammatory activation.

### 3.4 IFLT Reduced Recruitment of Recipient‐Derived Mono‐c1 via ANXA1‐FPR1 Axis

**FIGURE 4:**
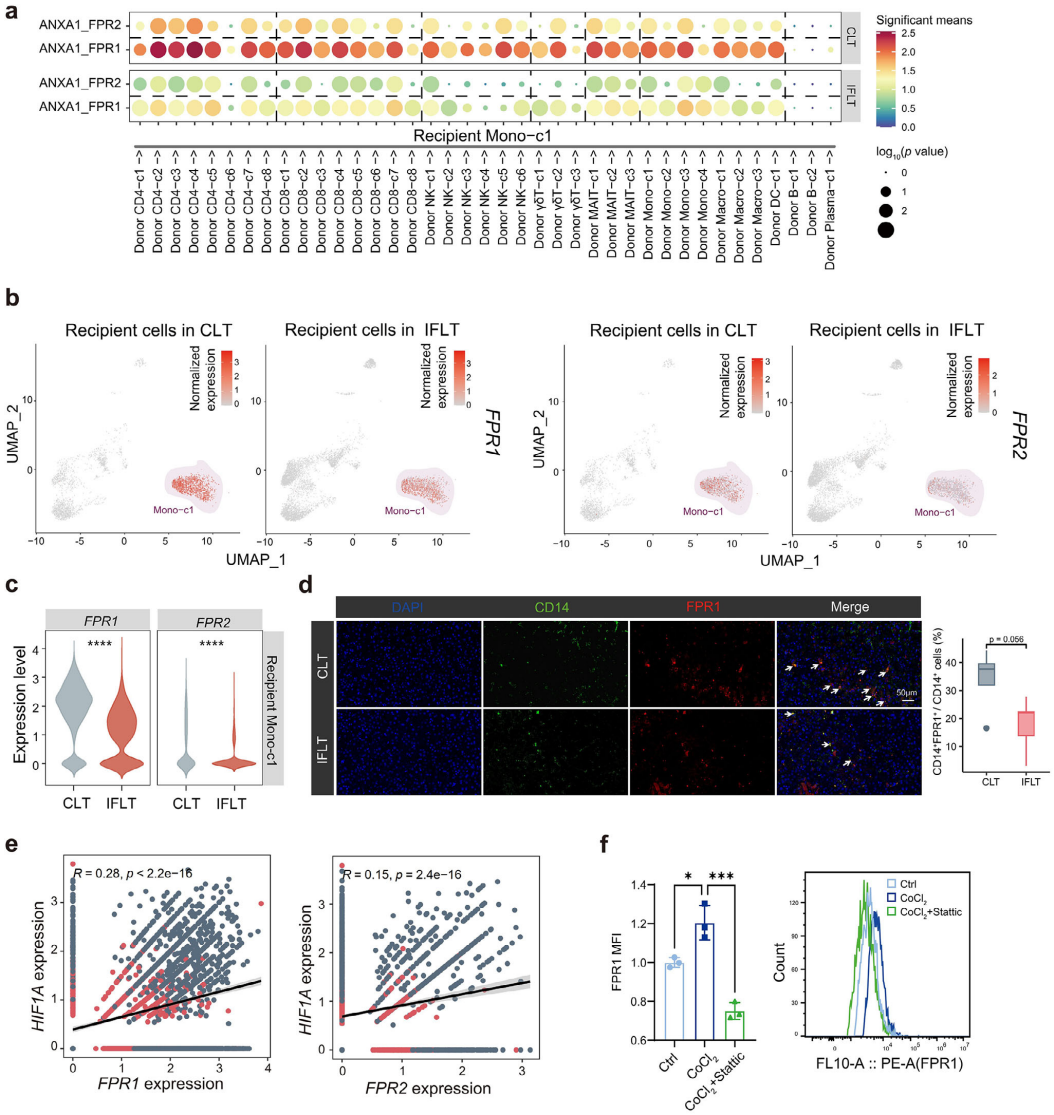
Attenuated ANXA1‐FPR1 signaling in IFLT reduces the infiltration of recipient‐derived Mono‐c1 cells. (a) The dot plot shows the interaction pairs with ANXA1 expressed on donor‐derived cells and FPR1/FPR2 expressed on recipient‐derived Mono‐c1. (b) UMAP plot shows the expression level of *FPR1/2* in recipient‐derived cells across CLT and IFLT patients. (c) Violin plots show *FPR1/2* expression levels in the recipient‐derived Mono‐c1 between two groups. (d) IF staining of PR grafts from CLT (*n* = 5) and IFLT (*n* = 5) patients showing expression of CD14 (green) and FPR1 (red). The white arrow indicates the positive cells with co‐expression of CD14 and FPR1. Scale bars, 50 µm. (e) The scatter plot shows the Pearson correlation between *HIF1A* and *FPR1/2* expression levels in the recipient‐derived Mono‐c1 from the single‐cell sequencing dataset. (f) Bar plots showing the expression of FPR1 in monocytes under control conditions, CoCl2 treatment, or CoCl_2_ plus the STAT3 inhibitor Stattic, measured by flow cytometry (*n* = 3 per group). Representative histogram plots are shown to the right of the scatter plot. Monocytes were isolated from the peripheral blood of healthy donors. Ns, no significance; ^*^ *p* < 0.05; ^***^ *p* < 0.001; ^****^ *p* < 0.0001.

We observed a declined proportion of recipient‐derived Mono‐c1 in the IFLT group (Figure 2g), suggesting reduced monocyte infiltration compared with CLT. Cellchat analysis revealed markedly diminished activity of the chemotactic ligand‐receptor pair ANXA1‐FPR1/2, which is known to promote monocyte migration [33], between multiple cell populations and Mono‐c1 cells in IFLT (Figure 4a; Figure S6a). Consistently, FPR1 expression was significantly reduced in recipient‐derived Mono‐c1 cells from IFLT grafts (Figure 4b,c), and IF staining confirmed lower FPR1 levels at PR (Figure 4d). A similar trend was observed in peripheral blood monocytes, which showed decreased FPR1 expression in IFLT at 2 h post‐reperfusion (Figure S6b).

Given the pronounced hypoxic conditions in CLT grafts, we hypothesized that the elevated FPR1 expression in CLT monocytes is regulated by the STAT3‐HIF‐1α signaling axis. Supporting this model, we observed a significant positive correlation between *HIF1A* and *FPR1* expression (Figure 4e). To further validate this mechanism, PBMC‐derived monocytes were stimulated with CoCl_2_, with or without a STAT3 inhibitor, to mimic hypoxia. Flow cytometry showed that CoCl_2_ robustly induced FPR1, whereas STAT3 inhibition markedly reduced FPR1 levels (Figure 4f). These results demonstrate that *FPR1* expression is upregulated under hypoxia in a STAT3‐HIF‐1α‐dependent manner. Additionally, IF staining indicated reduced ANXA1 expression in IFLT grafts (Figure S7c–e). Collectively, these findings demonstrate that by suppressing the STAT3‐HIF‐1α signaling axis, IFLT downregulates *FPR1* expression in monocytes, which in turn weakens ANXA1‐FPR1‐mediated chemotactic recruitment and reduces recipient‐derived monocyte infiltration into the graft.

### 3.5 Reduced Inflammasome Activation and Immunogenicity in IRI‐Sensitive Monocytes/Macrophages in IFLT versus CLT Grafts

Monocytes and macrophages have been identified as the major innate immune cells contributing to the pathogenesis of hepatic IRI in experimental models [34]. We then performed a comprehensive examination of both macrophages and monocytes, and discovered a significant downregulation of inflammasome‐associated genes in IFLT grafts, including *NLRP3* [34], *DDX3X* [35], and *GBP2* [36] (Figure S8a,b). Moreover, the activity of the “inflammasome complex assembly” pathway was reduced in IFLT grafts, particularly within Macro‐c1, Macro‐c2, and Mono‐c1 (Figure S8c). In PR liver biopsy samples, donor‐derived Macro‐c1, Macro‐c2, and Mono‐c1, as well as recipient‐derived Mono‐c1, constituted 88.1% of myeloid cells (Figure S8d). Downregulation of inflammasome‐associated genes, including *NLRP3*, *DDX3X*, and *GBP2*, was observed in donor‐derived Macro‐c1, Macro‐c2, Mono‐c1, and recipient‐derived Mono‐c1 cells in IFLT versus CLT grafts (Figure S8e). Assembly of the NOD‐like receptor thermal protein domain‐associated protein 3 (NLRP3)‐mediated inflammasome leads to the release of interleukin‐1β (IL‐1β) and cell pyroptosis [37]. Immunofluorescence staining confirmed that the expression of IL‐1β and NLRP3 was decreased in monocytes and macrophages in IFLT versus CLT grafts (Figure S8f–i).

Moreover, in studies across all cell types, inflammatory stimuli led to a notable increase in the expression of major histocompatibility complex (MHC) class I molecules [38] encoded by immunogenicity genes associated with acute liver transplant rejection [39]. In our analysis, donor‐derived Macro‐c1, Macro‐c2, and Mono‐c1 and recipient‐derived Mono‐c1 exhibited lower expression of classical MHC class I genes, including *HLA‐A*, *HLA‐B*, and *HLA‐C*, in IFLT versus CLT grafts (Figure S8j).

Considering that IFLT markedly improves the hypoxic microenvironment in the graft, we further hypothesized that IFLT might modulate key inflammasome‐related molecules in monocytes, including IL‐1β, NLRP3, inflammatory cytokines (IL‐6 and TNF‐α), and MHC‐I, through the STAT3‐HIF‐1α pathway. Flow cytometry of recipient peripheral blood monocytes at PR revealed significantly lower expression of IL‐1β, IL‐6, and TNF‐α in the IFLT group, whereas NLRP3 and MHC‐I levels showed no significant differences (Figure S9a–e). To mechanistically validate the hypoxia‐driven regulation of these molecules, CoCl_2_‐induced hypoxia in healthy‐donor monocytes robustly increased NLRP3, IL‐1β, IL‐6, TNF‐α, and MHC‐I expression, while STAT3 inhibition markedly attenuated these effects (Figure S9f–j).

Collectively, these data suggest that IFLT mitigates the graft's hypoxia and, through suppression of the STAT3‐HIF‐1α axis, dampens downstream inflammasome activation, pro‐inflammatory gene, and immunogenic gene expression in myeloid cells.

### 3.6 Enhanced “Porphyrin and Heme Metabolism” Activity in IRI‐Sensitive Monocytes/Macrophages in IFLT versus CLT Grafts

**FIGURE 5:**
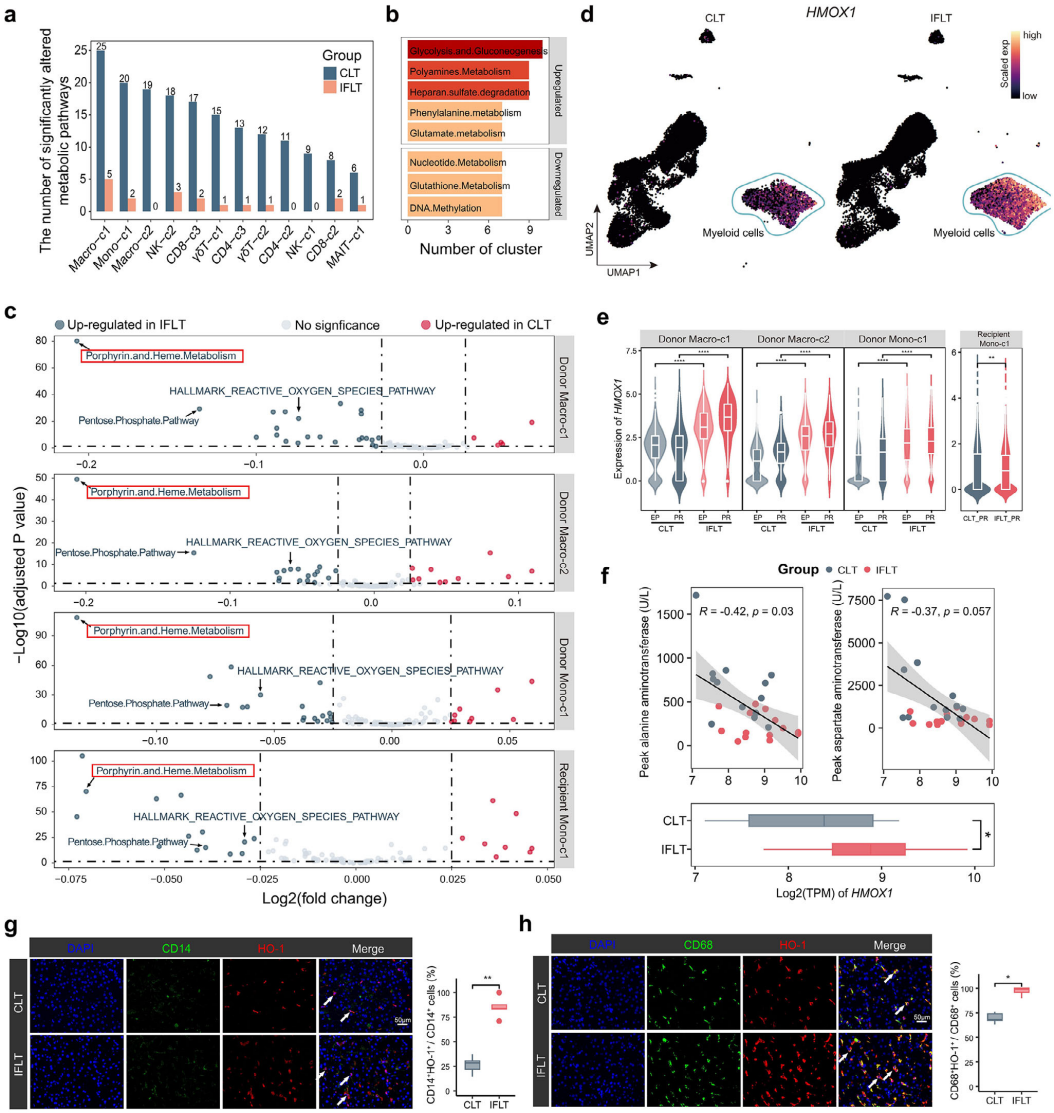
Metabolic features in monocytes and macrophages across CLT and IFLT grafts. (a) Bar plot shows the number of significant metabolic pathways (PR vs EP) in 12 donor‐derived IRIs‐icells clusters across CLT and IFLT patients. (b) Bar plot shows the significant common downregulated and upregulated metabolic pathways across different cell clusters. (c) Volcano plot shows differentially expressed metabolic pathways in monocytes and macrophages in PR samples (CLT vs IFLT). (d) UMAP plots show the expression level of *HMOX1* in CLT and IFLT grafts. (e) Violin plots show *HMOX1* expression levels in donor‐ and recipient‐derived cell clusters across EP and PR samples from CLT and IFLT patients. (f) Scatter plot shows the Pearson correlation between *HMOX1* expression levels in grafts and liver enzyme levels in peripheral blood post‐transplantation. A bar plot shows expression levels of HMOX1 across CLT and IFLT patients. (g), (h) IF staining of CLT and IFLT grafts shows the HO‐1 expression levels in monocytes (g) and macrophages (h) (CD68 and CD14, *n* = 5 per group). Ns, no significance; ^*^ *p* < 0.05; ^**^ *p* < 0.01; ^***^ *p* < 0.001; ^****^ *p* < 0.0001.

In our previous study [20], we observed significant metabolic reprogramming in CLT grafts. In contrast, the grafts maintained a stable metabolism throughout the IFLT. However, whether and how immune cells are regulated by metabolic reprogramming during IRI remains unclear. We then compared the metabolic dynamics (PR vs. EP phases) of donor‐derived IRIs‐icells between CLT and IFLT grafts. Significant metabolic fluctuations were identified in IRIs‐icells of CLT grafts, particularly in Macro‐c1 (25 pathways), Mono‐c1 (20 pathways), and Macro‐c2 (19 pathways) (Figure 5a). Notably, pathways such as “Glycolysis and Gluconeogenesis,” “Polyamines Metabolism,” and “Heparan sulfate degradation” were upregulated, whereas “Nucleotide Metabolism” and “Glutathione Metabolism” were downregulated upon reperfusion compared with preservation in CLT grafts (Figure 5b). These results demonstrate significant metabolic reprogramming in donor‐derived IRIs‐icells of CLT grafts. In contrast, donor‐derived IRIs‐icells maintained metabolic homeostasis in IFLT grafts.

Next, we compared the metabolic effects of IFLT on donor‐derived IRIs‐icells at PR. The results revealed that, in the majority of clusters, “HALLMARK_HYPOXIA” was downregulated in the IFLT group, further supporting our findings that IFLT effectively mitigates graft hypoxia (Figure S10). Interestingly, in donor‐derived Mono‐c1, Macro‐c1, Macro‐c2, and even recipient‐derived Mono‐c1, “Porphyrin and Heme Metabolism” was markedly upregulated in IFLT versus CLT grafts (Figure 5c). Heme oxygenase‐1 (HO‐1), encoded by *HMOX1*, is a pivotal enzyme in heme degradation [40]. In our study, we found that *HMOX1* expression was confined to the myeloid subsets (Figure 5d). A consistent increase in *HMOX1* expression was observed in donor‐derived Mono‐c1, Macro‐c1, and Macro‐c2, as well as recipient‐derived Mono‐c1 in IFLT versus CLT grafts (Figure 5e). Moreover, we observed a negative correlation between *HMOX1* expression and post‐transplant peak serum alanine aminotransferase level (Figure 5f). IF staining demonstrated significant expression of HO‐1 in the CD14 and CD68 populations, with markedly increased expression in IFLT versus CLT grafts (Figure 5g,h). Furthermore, we revealed a negative correlation between “Porphyrin and Heme Metabolism” and “Inflammasome complex assembly” in these clusters (Figure S11a). A negative correlation was also observed between *HMOX1* and *IL1B* expression in donor‐derived Macro‐c1, Macro‐c2, and Mono‐c1 (Figure S11b). These results suggest that enhanced HO‐1 activity in IRI‐sensitive monocyte and macrophage clusters is the major protective metabolic regulation in IFLT grafts.

### 3.7 Reduced Pro‐Rejection but Enhanced Pro‐Tolerance Adaptive Immunity in IFLT versus CLT Recipients

**FIGURE 6:**
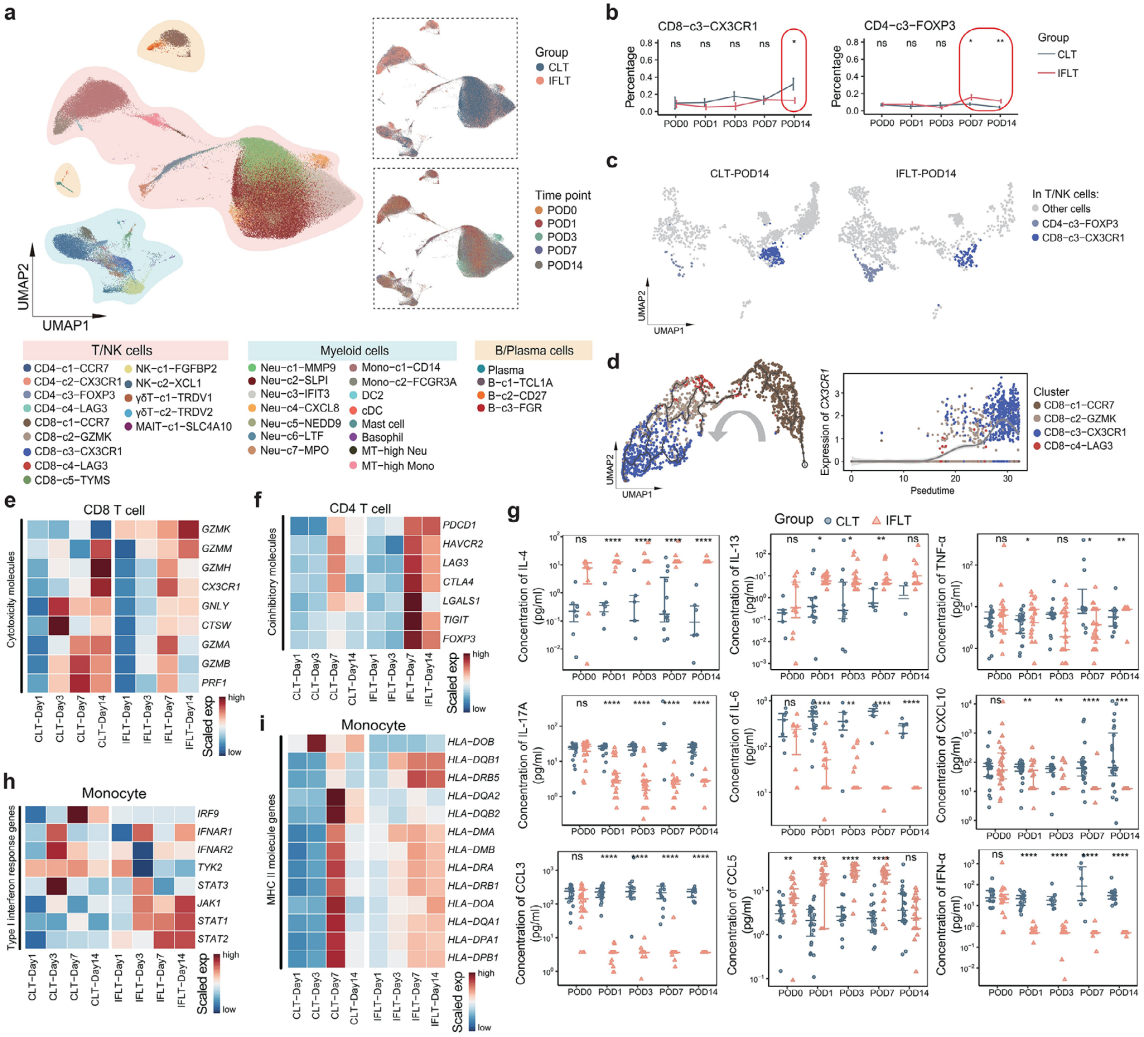
Downregulation of Teff/Th1/Th17 responses and upregulation of Treg/Th2 responses in IFLT versus CLT recipients. (a) The UMAP plot shows single cells from peripheral blood collected at different time points post‐transplantation. (b) Line plots show the cell proportions of CD8‐c3‐CX3CR1 and CD4‐c3‐FOXP3 within T cells (mean ± SEM). (c) UMAP plot shows CD8‐c3‐CX3CR1 and CD4‐c3‐FOXP3 cell clusters in peripheral blood samples on POD 14 across CLT and IFLT recipients. (d) UMAP plots show the developmental trajectory of CD8^+^ T cells in peripheral blood samples using Monocle3 (left panel). The dot plot shows the expression level of *CX3CR1* in CD8^+^ T cells along pseudotime (right panel). (e, f) Heatmap shows the average expression levels of cytotoxicity‐related genes in CD8^+^ T cells (e) and coinhibitory‐related genes in CD4^+^ T cells (f). (g) Jitter point plots show the serum levels of nine cytokines at different time points post‐transplantation across CLT (*n* = 30) and IFLT (*n* = 30) recipients. (h), (i) Heatmap shows the average expression levels of genes related to type I interferon response (h) and MHC II molecules (i) in monocytes at various time points post‐transplantation. Ns, no significance; ^*^ *p* < 0.05; ^**^ *p* < 0.01; ^***^ *p* < 0.001; ^****^ *p* < 0.0001.

To elucidate the impact of IFLT on systemic immunity, we generated a scRNA‐seq atlas of peripheral blood mononuclear cells (PBMC) from CLT and IFLT recipients. After stringent quality control, we obtained 165,387 immune cells, including three major clusters and 33 subclusters (Figure 6a; Figure S12a–d). CD8^+^ effector T cells (Teff) are the key effector cells mediating allograft rejection, whereas CD4^+^ regulatory T cells (Tregs) are required to maintain allograft tolerance [41, 42, 43]. We first examined the composition and transcription of adaptive immune cells. Cell composition analysis revealed a significant reduction in the number of CD8^+^ Teff (CD8‐c3‐CX3CR1) on POD 14 in IFLT versus CLT recipients (Figure 6b,c; Figure S12e). Pseudo‐time trajectory analysis showed a developmental path from naïve CD8^+^ T cells (CD8‐c1‐CCR7) to Teff, with increased *CX3CR1* expression (Figure 6d). Moreover, CD8^+^ T cells in IFLT recipients had lower levels of cytotoxic genes (such as *GZMB*, *GNLY*, and *PRF1*) than those in CLT recipients (Figure 6e). In addition, a higher proportion of Treg (CD4‐c3‐FOXP3) with augmented expression of inhibitory genes (such as *TIGIT*, *FOXP3*, and *CTLA4*) in CD4^+^ T cells was observed on POD 7 and 14 in IFLT versus CLT recipients (Figure 6b,f). There was no significant difference in the proportion of other clusters between the IFLT and CLT recipients.

Compared to CLT recipients, IFLT recipients exhibited reduced Th1 (TNF‐α) and Th17 (IL‐17A and IL‐6) cytokine levels, but elevated Th2 cytokine (IL‐4 and IL‐13) levels (Figure 6g). In addition, it has been reported that C‐X‐C motif chemokine 10 (CXCL10) and C‐C motif chemokine ligand (CCL3) promote the Th1 response [44, 45], whereas CCL5 induces Th2 polarization [46]. Consistent with these findings, we observed reduced CXCL10/CCL3 and increased CCL5 serum levels in IFLT versus CLT recipients (Figure 6g). The results for the other cytokine/chemokine are shown in Figure S13.

Taken together, the scRNA‐seq and multi‐cytokine analyses of the peripheral blood demonstrated reduced pro‐rejection but enhanced pro‐tolerance adaptive immunity in IFLT versus CLT recipients.

### 3.8 Postponed Type I Interferon Responses and Reduced MHC II Molecule Expression in Peripheral Monocytes in IFLT versus CLT Recipients

Finally, we conducted a comprehensive analysis of peripheral innate immunity. Cell proportion analysis revealed no significant difference in neutrophil and monocyte composition between the two groups (Figure S12f). Gene profile changes (each time point vs. POD 0) in monocytes in the CLT group occurred in the first week post‐transplantation. In contrast, the gene profile changes in the IFLT group were postponed until the second week post‐transplantation (Figure S12g). These DEGs in monocytes in both CLT and IFLT were enriched in type I interferon (IFN) responses (*STAT3*, *IRF9*, *IFNAR1*, *IFNAR2*, and *TYK2*) and MHC II molecules (*HLA‐DMA* and *HLA‐DMB*) (Figure 6h,i). We also observed a significant increase in serum IFN‐α level on POD 7 in CLT versus IFLT recipients (Figure 6g). Interestingly, the significant upregulation of almost all MHC II molecules in monocytes occurred selectively on POD 7 in CLT recipients (Figure 6i). In contrast, the expression of only two MHC II molecules, *HLA‐DRB5* and *HLA‐DQB1*, was moderately upregulated in monocytes from POD 7 to 14 in IFLT recipients (Figure 6i). The gene profile dynamics of neutrophils were comparable between the two groups (Figure S12g–j). Collectively, postponed type I interferon responses and reduced MHC II molecule expression in monocytes represent characteristic impacts of the ischemia‐free procedure on peripheral innate immunity in human liver transplantation.

## 4 Discussion

During IRI, various immune cells and pathways are activated and actively involved in the pathogenesis of liver injury and transplant outcomes [47]. In the current study, we documented significant impacts of a novel ischemia‐free procedure designed to avoid graft ischemia on local and systemic immunity in human liver transplantation.

Neutrophils are the earliest infiltrating pivotal cells that cause parenchymal damage in hepatic IRI [9]. In this study, neutrophil infiltration was lower in IFLT grafts than in CLT grafts. A recent study showed that neutrophils can be “washed out” during ex situ NMP [48]. It is still unknown whether the reduced neutrophil infiltration in IFLT grafts is attributable to this “washed out” effect or compromised recruitment. Studies have shown a key role for NETs formation in mouse hepatic IRI, and serum NETs markers are correlated with graft injury in humans [26, 49]. Moreover, DNA released from NETs is a major source of danger‐associated molecular patterns (DAMP), which activate innate immune cells and trigger sterile inflammation. Targeting NETs is a potentially effective treatment [49]. In the current study, we showed that the application of our novel IFLT technique leads to a substantial reduction in intra‐graft NETs formation.

By comparing the IFLT and CLT immune cell atlases, 12 donor‐derived and four recipient‐derived IRIs‐icells clusters were identified with common gene transcriptional regulation, which represents their common responses to metabolic reprogramming during graft IRI. Interestingly, “Porphyrin and Heme Metabolism” was the most markedly upregulated pathway, with enhanced HO‐1 expression in these clusters of IFLT versus CLT grafts. Previous studies have shown that heme inactivates Bach1, a transcription repressor, thereby enabling the activation of Maf recognition elements (MAREs) containing genes, such as *HMOX1* [50, 51]. Because IFLT employs continuous blood perfusion enriched with hemoglobin to preserve graft stability during transplantation, this heme‐rich environment likely enhances *HMOX1* expression in monocytes and macrophages. Nakamura et al. showed that low HO‐1 levels in macrophages of reperfused human grafts correlated with inferior clinical outcomes [52]. Studies in mice have demonstrated that HO‐1 expression in both donor‐ and recipient‐derived macrophages is crucial for IRI pathogenesis [52, 53]. HO‐1 overexpression in macrophages ameliorates liver IRI in mice [54]. We documented enhanced HO‐1 expression not only in specific macrophage clusters (donor‐derived Macro‐c1 and Macro‐c2) but also in specific monocyte clusters (donor‐derived and recipient‐derived Mono‐c1), in IFLT versus CLT grafts. These results suggest that HO‐1 expression is suppressed in specific IRI‐sensitive monocyte and macrophage clusters in CLT, leading to profound graft IRI, whereas IFLT can maintain high HO‐1 expression in these clusters, thus protecting the grafts from IRI.

Reports have revealed that during IRI, circulating monocytes are recruited to the inflammation site [55] and then differentiate and replenish the macrophage pool, thereby promoting sterile inflammation [56]. We documented a significantly reduced recruitment of recipient‐derived Mono‐c1 in IFLT versus CLT grafts via the ANXA1‐FPR1 axis. Interestingly, we found that IFLT attenuated graft hypoxia through sustained oxygenated perfusion, thereby reducing STAT3‐HIF‐1α‐mediated induction of *FPR1*. This, in turn, suppressed the ANXA1‐FPR1 chemotactic axis and diminished the infiltration of recipient‐derived Mono‐c1 cells. Collectively, these results suggest that the migration and activation of recipient‐derived circulating monocytes occur very early after graft reperfusion, serving as key players in graft IRI in human liver transplantation.

Understanding how local graft IRI regulates systemic immune responses after liver transplantation is of great interest in developing therapies against allograft rejection and remote organ injuries [5, 57]. Our scRNA‐seq analysis demonstrated increased expression of almost all MHC II molecules in monocytes, selectively on POD 7 in CLT rather than in IFLT recipients. Intriguingly, significantly reduced peripheral CD8^+^ Teff composition on POD 14 but increased Treg composition on POD 7 and 14 were documented in IFLT versus CLT recipients. Previous studies have shown that the cytotoxic capacity of CD8^+^ Teff contributes to allograft rejection [58, 59], whereas Tregs promote allograft tolerance by suppressing the activation of other T cells [60]. Moreover, cytokine/chemokine profiling showed reduced Th1/Th17 but enhanced Th2 responses in IFLT versus CLT recipients. Previous studies have shown that Th1 and Th17 cells are critical drivers of allograft rejection [61, 62], whereas Th2 cells contribute to transplant tolerance [63]. Collectively, these results suggest that systemic immunity switches from allograft rejection to transplant tolerance in IFLT versus CLT recipients.

Our study also has limitations. First, liver biopsies were not feasible at long‐term postoperative timepoints, which restricted our ability to assess the persistence or replacement of donor‐derived cells within the graft and to fully characterize the establishment of immune homeostasis. Second, non‐immune parenchymal cells were not captured in our dataset, limiting our capacity to evaluate their important roles within the graft's immune regulatory network during the acute post‐transplant response. Future studies incorporating longitudinal graft sampling and comprehensive profiling of both immune and non‐immune compartments will be essential to fully elucidate IRI mechanisms.

In conclusion, our data demonstrate that IFLT can tip the balance from pro‐ to anti‐ inflammation locally, and from pro‐ to anti‐ rejection systemically. Moreover, our study described a comprehensive and precise roadmap of local and systemic immunity during graft IRI, providing a unique resource for understanding the mechanisms of graft IRI and allograft rejection in human liver transplantation.

## Author Contributions

Z.G. conceived of the study. Z.G., X.H., J.B., and Y.G. supervised this study. T.L., S.H., D.Y., S.Z., D.Z., L.W., H.L., R.L., X.T., Y.W., J.Z., T.W., Z.J., Y.J., T.F., Y.L., and X.L. contributed to the sample and information collection and data generation. Y.G. and D.Y. supervised the Luminex cytokine assays. Z.G. and X.H. supervised the bioinformatics data processing and analysis. T.L. and S.H. performed the bioinformatics data analysis. S.C. and S.Z. performed IF, IHC, and cell culture assays. D.L., H.C., J.X., R.M., Y.L., Y.Z., and T.R. assisted with the data processing. Z.G., T.L., S.H., X.H., Y.G., L.W., Y.T., M.C., Q.Z, A.H., W.J., Y.M., D.W., X.Z., A.S., Y.Z., K.M., and S.G.T. contributed to the data interpretation. Z.G., X.H., J.B., and Y.G. wrote and revised the manuscript, and all the co‐authors reviewed the manuscript.

## Funding

This study was supported by grants as follows: the National Natural Science Foundation of China (82170663, 82370664, 82300744, W2511088 and 82525012), the Guangdong Provincial Key Laboratory Construction Projection on Organ Donation and Transplant Immunology (2023B1212060020), Guangdong Provincial International Cooperation Base of Science and Technology (Organ Transplantation) (2020A0505020003), Science and Technology Program of Guangdong (2024B1515040011), and the Natural Science Foundation of Guangdong Province (2024A1515013030). This study was also supported by China Organ Transplantation Development Foundation.

## Ethical Approval Statement

All the research was conducted in accordance with the Declaration of Helsinki and Istanbul. Written consent was obtained from all subjects. The study protocol was approved by the Ethics Committee of the First Affiliated Hospital of Sun Yat‐Sen University ([2018]255 and [2019]037).

## Conflicts of Interest

The authors declare no conflicts of interest.

## Supporting information




**Supporting File 1**: advs73711‐sup‐0001‐SuppMat.docx.

**Supporting File 2**: advs73711‐sup‐0002‐TablesS1‐S6.xlsx.

## Data Availability

All the raw sequence data of scRNA‐seq have been deposited in the National Genomics Data Center (http://bigd.big.ac.cn/gsa‐human) with the accession numbers (HRA005028 and HRA008371). All the related codes and data analysis scripts are available at https://github.com/sysu‐Max/IFLT‐analysis.

## References

[1] J. E. Murray , “Human Organ Transplantation: Background and Consequences,” Science 256, no. 5062 (1992): 1411–1416, 10.1126/science.1604314. 1604314

[2] D. P. Foley , “Comparing Preservation Solutions For Static Cold Storage In Donation After Circulatory Death Liver Transplantation,” Liver Transplantation 28, no. 9 (2022): 1423–1424, 10.1002/lt.26528. 35706126

[3] T. Ito , B. V. Naini , D. Markovic , et al., “Ischemia‐Reperfusion Injury And Its Relationship With Early Allograft Dysfunction In Liver Transplant Patients,” American Journal of Transplantation 21, no. 2 (2021): 614–625, 10.1111/ajt.16219. 32713098

[4] H. Zhao , A. Alam , A. P. Soo , A. J. T. George , and D. Ma , “Ischemia‐Reperfusion Injury Reduces Long Term Renal Graft Survival: Mechanism and Beyond,” EBioMedicine 28 (2018): 31–42, 10.1016/j.ebiom.2018.01.025. 29398595 PMC5835570

[5] J. Ochando , F. Ordikhani , P. Boros , and S. Jordan , “The Innate Immune Response To Allotransplants: Mechanisms And Therapeutic Potentials,” Cellular & Molecular Immunology 16, no. 4 (2019): 350–356, 10.1038/s41423-019-0216-2. 30804476 PMC6462017

[6] K. J. Dery , S. Yao , B. Cheng , and J. W. Kupiec‐Weglinski , “New Therapeutic Concepts Against Ischemia‐Reperfusion Injury In Organ Transplantation,” Expert Review of Clinical Immunology 19, no. 10 (2023): 1205–1224, 10.1080/1744666X.2023.2240516. 37489289 PMC10529400

[7] M. H. Oberbarnscheidt , Q. Zeng , Q. Li , et al., “Non‐Self Recognition By Monocytes Initiates Allograft Rejection,” Journal of Clinical Investigation 124, no. 8 (2014): 3579–3589, 10.1172/JCI74370. 24983319 PMC4109551

[8] K. C. Tatham , K. P. O'Dea , R. Romano , et al., “Intravascular Donor Monocytes Play A Central Role In Lung Transplant Ischaemia‐Reperfusion Injury,” Thorax 73, no. 4 (2018): 350–360, 10.1136/thoraxjnl-2016-208977. 28389600 PMC5870457

[9] H. Hirao , K. Nakamura , and J. W. Kupiec‐Weglinski , “Liver Ischaemia–Reperfusion Injury: A New Understanding Of The Role Of Innate Immunity,” Nature Reviews Gastroenterology & Hepatology 19, no. 4 (2022): 239–256, 10.1038/s41575-021-00549-8. 34837066

[10] X. Yang , D. Lu , R. Wang , et al., “Single‐Cell Profiling Reveals Distinct Immune Phenotypes That Contribute To Ischaemia‐Reperfusion Injury After Steatotic Liver Transplantation,” Cell Proliferation 54, no. 10 (2021): 13116, 10.1111/cpr.13116. PMC8488562 34469018

[11] C. Sun , L. Li , D. Li , and Z. Wang , “Discovery of Endothelial–Monocyte Crosstalk in Ischemic‐Reperfusion Injury Following Liver Transplantation Based on Integration of Single‐Cell RNA and Transcriptome RNA Sequencing,” Journal of Cellular and Molecular Medicine 29, no. 4 (2025): 70336, 10.1111/jcmm.70336. PMC11850096 39993960

[12] Y. Shan , D. Qi , L. Zhang , et al., “Single‐Cell Rna‐Seq Revealing The Immune Features Of Donor Liver During Liver Transplantation,” Frontiers In Immunology 14 (2023): 1096733, 10.3389/fimmu.2023.1096733. 36845096 PMC9945228

[13] X. Li , S. Li , B. Wu , et al., “Landscape of Immune Cells Heterogeneity in Liver Transplantation by Single‐Cell RNA Sequencing Analysis,” Frontiers in Immunology 13 (2022): 890019, 10.3389/fimmu.2022.890019. 35619708 PMC9127089

[14] R. Wang , X. Peng , Y. Yuan , et al., “Dynamic Immune Recovery Process After Liver Transplantation Revealed By Single‐Cell Multi‐Omics Analysis,” Innovation 5, no. 3 (2024): 100599, 10.1016/j.xinn.2024.100599. 38510071 PMC10952083

[15] Z. Wu , D. Liu , Y. Ou , et al., “Mechanism And Endoscopic‐Treatment‐Induced Evolution Of Biliary Non‐Anastomotic Stricture After Liver Transplantation Revealed By Single‐Cell Rna Sequencing,” Clinical and Translational Medicine 14, no. 3 (2024): 1622, 10.1002/ctm2.1622. PMC10938070 38481381

[16] X. He , Z. Guo , Q. Zhao , et al., “The First Case Of Ischemia‐Free Organ Transplantation In Humans: A Proof Of Concept,” American Journal of Transplantation 18, no. 3 (2018): 737–744, 10.1111/ajt.14583. 29127685

[17] X. He , G. Chen , Z. Zhu , et al., “The First Case Of Ischemia‐Free Kidney Transplantation In Humans,” Frontiers in Medicine 6 (2019): 276, 10.3389/fmed.2019.00276. 31921864 PMC6917615

[18] S. Yin , J. Rong , Y. Chen , et al., “Transplantation Of A Beating Heart: A First In Man,” The Lancet Regional Health–Western Pacific 23 (2022): 100449, 10.1016/j.lanwpc.2022.100449. 35465045 PMC9019404

[19] Z. Guo , Q. Zhao , Z. Jia , et al., “A Randomized‐Controlled Trial Of Ischemia‐Free Liver Transplantation For End‐Stage Liver Disease,” Journal of hepatology 79, no. 2 (2023): 394–402, 10.1016/j.jhep.2023.04.010. 37086919

[20] Z. Guo , J. Xu , S. Huang , et al., “Abrogation Of Graft Ischemia‐Reperfusion Injury In Ischemia‐Free Liver Transplantation,” Clinical and Translational Medicine 12, no. 4 (2022): 546, 10.1002/ctm2.546. PMC9042797 35474299

[21] Z. Guo , Q. Zhao , S. Huang , et al., “Ischaemia‐Free Liver Transplantation In Humans: A First‐In‐Human Trial,” The Lancet Regional Health–Western Pacific 16 (2021): 100260, 10.1016/j.lanwpc.2021.100260. 34590063 PMC8406025

[22] S. He , L. H. Wang , Y. Liu , et al., “Single‐Cell Transcriptome Profiling Of An Adult Human Cell Atlas Of 15 Major Organs,” Genome Biology 21, no. 1 (2020): 294, 10.1186/s13059-020-02210-0. 33287869 PMC7720616

[23] T. Stuart , A. Butler , P. Hoffman , et al., “Comprehensive Integration of Single‐Cell Data,” Cell 177, no. 7 (2019): 1888–1902, 10.1016/j.cell.2019.05.031. 31178118 PMC6687398

[24] H. Heaton , A. M. Talman , A. Knights , et al., “Souporcell: Robust Clustering Of Single‐Cell Rna‐Seq Data By Genotype Without Reference Genotypes,” Nature Methods 17, no. 6 (2020): 615–620, 10.1038/s41592-020-0820-1. 32366989 PMC7617080

[25] A. M. Newman , C. L. Liu , M. R. Green , et al., “Robust Enumeration Of Cell Subsets From Tissue Expression Profiles,” Nature Methods 12, no. 5 (2015): 453–4573, 10.1038/nmeth.3337. 25822800 PMC4739640

[26] H. Hirao , H. Kojima , K. J. Dery , et al., “Neutrophil Ceacam1 Determines Susceptibility To Netosis By Regulating The S1PR2/S1PR3 Axis In Liver Transplantation,” Journal of Clinical Investigation 133, no. 3 (2023): 162940, 10.1172/JCI162940. PMC9888387 36719377

[27] X. Liu , T. Arfman , K. Wichapong , C. P. M. Reutelingsperger , J. Voorberg , and G. A. F. Nicolaes , “PAD4 Takes Charge During Neutrophil Activation: Impact Of PAD4 Mediated Net Formation On Immune‐Mediated Disease,” Journal of Thrombosis and Haemostasis 19, no. 7 (2021): 1607–1617, 10.1111/jth.15313. 33773016 PMC8360066

[28] J. T. Dancey , K. A. Deubelbeiss , L. A. Harker , and C. A. Finch , “Neutrophil Kinetics In Man,” Journal of Clinical Investigation 58, no. 3 (1976): 705–715, 10.1172/JCI108517. 956397 PMC333229

[29] S. Basu , G. Hodgson , M. Katz , and A. R. Dunn , “Evaluation Of Role Of G‐Csf In The Production, Survival, And Release Of Neutrophils From Bone Marrow Into Circulation,” Blood 100, no. 3 (2002): 854–861, 10.1182/blood.V100.3.854. 12130495

[30] Q. Xu , J. Briggs , S. Park , et al., “Targeting STAT3 Blocks Both HIF‐1 And Vegf Expression Induced By Multiple Oncogenic Growth Signaling Pathways,” Oncogene 24, no. 36 (2005): 5552–5560, 10.1038/sj.onc.1208719. 16007214

[31] J. E. Jung , H. G. Lee , I. H. Cho , et al., “STAT3 Is A Potential Modulator Of HIF‐1‐Mediated Vegf Expression In Human Renal Carcinoma Cells,” The FASEB Journal 19, no. 10 (2005): 1296–1298, 10.1096/fj.04-3099fje. 15919761

[32] H.‐Y. Fang , R. Hughes , C. Murdoch , et al., “Hypoxia‐Inducible Factors 1 And 2 Are Important Transcriptional Effectors In Primary Macrophages Experiencing Hypoxia,” Blood 114, no. 4 (2009): 844–859, 10.1182/blood-2008-12-195941. 19454749 PMC2882173

[33] S. Ernst , C. Lange , A. Wilbers , V. Goebeler , V. Gerke , and U. Rescher , “An Annexin 1 N‐Terminal Peptide Activates Leukocytes by Triggering Different Members of the Formyl Peptide Receptor Family,” The Journal of Immunology 172, no. 12 (2004): 7669–7676, 10.4049/jimmunol.172.12.7669. 15187149

[34] S. Yue , J. Zhu , M. Zhang , et al., “The Myeloid Heat Shock Transcription Factor 1/Β‐Catenin Axis Regulates Nlr Family, Pyrin Domain‐Containing 3 Inflammasome Activation In Mouse Liver Ischemia/Reperfusion Injury,” Hepatology 64, no. 5 (2016): 1683–1698, 10.1002/hep.28739. 27474884 PMC5074868

[35] P. Samir , S. Kesavardhana , D. M. Patmore , et al., “DDX3X Acts As A Live‐Or‐Die Checkpoint In Stressed Cells By Regulating NLRP3 Inflammasome,” Nature 573, no. 7775 (2019): 590–594, 10.1038/s41586-019-1551-2. 31511697 PMC6980284

[36] R. Finethy , I. Jorgensen , A. K. Haldar , et al., “Guanylate Binding Proteins Enable Rapid Activation of Canonical and Noncanonical Inflammasomes in Chlamydia‐Infected Macrophages,” Infection and Immunity 83, no. 12 (2015): 4740–4749, 10.1128/IAI.00856-15. 26416908 PMC4645370

[37] K. V. Swanson , M. Deng , and J. P. Y. Ting , “The NLRP3 Inflammasome: Molecular Activation And Regulation To Therapeutics,” Nature Reviews Immunology 19, no. 8 (2019): 477–489, 10.1038/s41577-019-0165-0. PMC7807242 31036962

[38] A. J. Demetris , C. O. C. Bellamy , C. R. Gandhi , S. Prost , Y. Nakanuma , and D. B. Stolz , “Functional Immune Anatomy Of The Liver—As An Allograft,” American Journal of Transplantation 16, no. 6 (2016): 1653–1680, 10.1111/ajt.13749. 26848550

[39] S. K. So , J. L. Platt , N. L. Ascher , and D. C. Snover , “Increased Expression Of Class I Major Histocompatibility Complex Antigens On Hepatocytes In Rejecting Human Liver Allografts,” Transplantation 43, no. 1 (1987): 79–84, 10.1097/00007890-198701000-00018. 3541328

[40] M. D. Maines , “The Heme Oxygenase System: A Regulator Of Second Messenger Gases,” Annual Review of Pharmacology and Toxicology 37 (1997): 517–554, 10.1146/annurev.pharmtox.37.1.517. 9131263

[41] D. Kreisel , A. S. Krupnick , A. E. Gelman , et al., “Non‐Hematopoietic Allograft Cells Directly Activate CD8^+^ T Cells And Trigger Acute Rejection: An Alternative Mechanism Of Allorecognition,” Nature Medicine 8, no. 3 (2002): 233–239, 10.1038/nm0302-233. 11875493

[42] Y.‐S. Mederacke , M. Nienen , M. Jarek , et al., “T Cell Receptor Repertoires Within Liver Allografts Are Different To Those In The Peripheral Blood,” Journal of Hepatology 74, no. 5 (2021): 1167–1175, 10.1016/j.jhep.2020.12.014. 33347951

[43] K. J. Wood and S. Sakaguchi , “Regulatory T Cells In Transplantation Tolerance,” Nature Reviews Immunology 3, no. 3 (2003): 199–210, 10.1038/nri1027. 12658268

[44] G. Xanthou , C. E. Duchesnes , T. J. Williams , and J. E. Pease , “CCR3 Functional Responses Are Regulated By Both CXCR3 And Its Ligands CXCL9, CXCL10 And CXCL11,” European Journal of Immunology 33, no. 8 (2003): 2241–2250, 10.1002/eji.200323787. 12884299

[45] W. J. Karpus and K. J. Kennedy , “MIP‐1α And MCP‐1 Differentially Regulate Acute And Relapsing Autoimmune Encephalomyelitis As Well As Th1/Th2 Lymphoctye Differentiation,” Journal of Leukocyte Biology 62, no. 5 (1997): 681–687, 10.1002/jlb.62.5.681. 9365124

[46] Q. Zhang , J. Qin , L. Zhong , et al., “CCL5‐Mediated Th2 Immune Polarization Promotes Metastasis in Luminal Breast Cancer,” Cancer Research 75, no. 20 (2015): 4312–4321, 10.1158/0008-5472.CAN-14-3590. 26249173

[47] M. Zhang , Q. Liu , H. Meng , et al., “Ischemia‐Reperfusion Injury: Molecular Mechanisms And Therapeutic Targets,” Signal Transduction And Targeted Therapy 9, no. 1 (2024): 12, 10.1038/s41392-023-01688-x. 38185705 PMC10772178

[48] T. Hautz , S. Salcher , M. Fodor , et al., “Immune Cell Dynamics Deconvoluted By Single‐Cell Rna Sequencing In Normothermic Machine Perfusion Of The Liver,” Nature Communications 14, no. 1 (2023): 2285, 10.1038/s41467-023-37674-8. PMC10121614 37085477

[49] S. Zhang , Q. Zhang , F. Wang , et al., “Hydroxychloroquine Inhibiting Neutrophil Extracellular Trap Formation Alleviates Hepatic Ischemia/Reperfusion Injury By Blocking Tlr9 In Mice,” Clinical Immunology 216 (2020): 108461, 10.1016/j.clim.2020.108461. 32437924

[50] K. Ogawa , J. Sun , S. Taketani , et al., “Heme Mediates Derepression Of Maf Recognition Element Through Direct Binding To Transcription Repressor Bach1,” The EMBO Journal 20, no. 11 (2001): 2835–2843, 10.1093/emboj/20.11.2835. 11387216 PMC125477

[51] Y. Shan , R. W. Lambrecht , T. Ghaziani , S. E. Donohue , and H. L. Bonkovsky , “Role of Bach_‐1_ in Regulation of Heme Oxygenase_‐1_ in Human Liver Cells,” Journal of Biological Chemistry 279, no. 50 (2004): 51769–51774, 10.1074/jbc.M409463200. 15465821

[52] K. Nakamura , M. Zhang , S. Kageyama , et al., “Macrophage Heme Oxygenase‐_1_‐Sirt_1_‐_p53_ Axis Regulates Sterile Inflammation In Liver Ischemia‐Reperfusion Injury,” Journal of Hepatology 67, no. 6 (2017): 1232–1242, 10.1016/j.jhep.2017.08.010. 28842295 PMC5884687

[53] S. Kageyama , H. Hirao , K. Nakamura , et al., “Recipient HO_‐1_ inducibility is essential for posttransplant hepatic HO_‐1_ expression and graft protection: From bench‐to‐bedside,” American Journal of Transplantation 19, no. 2 (2019): 356–367, 10.1111/ajt.15043. 30059195 PMC6349504

[54] K. Nakamura , S. Kageyama , S. Yue , et al., “Heme Oxygenase_‐1_ Regulates Sirtuin_‐1_–Autophagy Pathway In Liver Transplantation: From Mouse To Human,” American Journal of Transplantation 18, no. 5 (2018): 1110–1121, 10.1111/ajt.14586. 29136322 PMC5910267

[55] H. H. Birdsall , D. M. Green , J. Trial , et al., “Complement C5A, TGF‐β1, and MCP‐1, in Sequence, Induce Migration of Monocytes Into Ischemic Canine Myocardium Within the First One to Five Hours After Reperfusion,” Circulation 95, no. 3 (1997): 684–692, 10.1161/01.CIR.95.3.684. 9024158

[56] J. Villar , L. Ouaknin , A. Cros , and E. Segura , “Monocytes Differentiate Along Two Alternative Pathways During Sterile Inflammation,” Embo Reports 24, no. 7 (2023): 56308, 10.15252/embr.202256308. PMC10328069 37191947

[57] C. Nastos , K. Kalimeris , N. Papoutsidakis , et al., “Global Consequences of Liver Ischemia/Reperfusion Injury,” Oxidative Medicine and Cellular Longevity 2014 (2014): 906965, 10.1155/2014/906965. 24799983 PMC3995148

[58] T. B. Strom , N. L. Tilney , C. B. Carpenter , and G. J. Busch , “Identity and Cytotoxic Capacity of Cells Infiltrating Renal Allografts,” New England Journal of Medicine 292, no. 24 (1975): 1257–1263, 10.1056/NEJM197506122922402. 1093024

[59] A. F. Malone , H. Wu , C. Fronick , R. Fulton , J. P. Gaut , and B. D. Humphreys , “Harnessing Expressed Single Nucleotide Variation and Single Cell RNA Sequencing To Define Immune Cell Chimerism in the Rejecting Kidney Transplant,” Journal of the American Society of Nephrology 31, no. 9 (2020): 1977–1986, 10.1681/asn.2020030326. 32669324 PMC7461682

[60] P. A. Taylor , C. J. Lees , and B. R. Blazar , “The Infusion Of Ex Vivo Activated And Expanded CD4^+^CD25^+^ Immune Regulatory Cells Inhibits Graft‐Versus‐Host Disease Lethality,” Blood 99, no. 10 (2002): 3493–3499, 10.1182/blood.V99.10.3493. 11986199

[61] W. J. Burlingham , R. B. Love , E. Jankowska‐Gan , et al., “IL‐17–Dependent Cellular Immunity To Collagen Type V Predisposes To Obliterative Bronchiolitis In Human Lung Transplants,” Journal of Clinical Investigation 117, no. 11 (2007): 3498–3506, 10.1172/JCI28031. 17965778 PMC2040314

[62] H. Fan , L.‐X. Li , D.‐D. Han , J.‐T. Kou , P. Li , and Q. He , “Increase Of Peripheral Th17 Lymphocytes During Acute Cellular Rejection In Liver Transplant Recipients,” Hepatobiliary & Pancreatic Diseases International 11, no. 6 (2012): 606–611. 23232631 10.1016/s1499-3872(12)60231-8

[63] C. Dai , F.‐N. Lu , N. Jin , et al., “Recombinant IL_‐33_ Prolongs Leflunomide‐Mediated Graft Survival By Reducing IFN‐Γ And Expanding Cd_4_ ^+^Fox_p3_ ^+^ T Cells In Concordant Heart Transplantation,” Laboratory Investigation 96, no. 8 (2016): 820–829, 10.1038/labinvest.2016.54. 27295346

