# Supplementary material for: Substantially Altered Local and Systemic Immunity in Ischemia‐Free Versus Conventional Liver Transplantation
Source: Adv Sci (Weinh). 2026 Jan 27;13(23):e02854. doi: 10.1002/advs.202502854 (PMC13104066; doi:10.1002/advs.202502854)
Supplement: Supplementary file 1 — Supporting File 1: advs73711‐sup‐0001‐SuppMat.docx. [file ADVS-13-e02854-s002.docx]

**­Substantially altered local and systemic immunity in ischemia-free versus conventional liver transplantation**

**Supplementary Information**

[Supplementary Methods 2](#_Toc215990997)

[Sample collection details 2](#_Toc215990998)

[Immunostaining assays 2](#_Toc215990999)

[Flow cytometry assays 3](#_Toc215991000)

[Monocytes isolation from PBMCs 3](#_Toc215991001)

[Western blot assays 4](#_Toc215991002)

[Cell culture under hypoxia-mimicking condition 4](#_Toc215991003)

[Estimation of infiltrated immune cell proportion using CIBERSORT 4](#_Toc215991004)

[Tissue dissociation and single-cell suspension preparations 5](#_Toc215991005)

[Single-cell library preparation 5](#_Toc215991006)

[Single-cell RNA sequencing (scRNA-seq) data processing 6](#_Toc215991007)

[Doublet identification and cell clustering 6](#_Toc215991008)

[Identification of cell identities in the peripheral blood mixture 7](#_Toc215991009)

[Identification of cells from donors and recipients 8](#_Toc215991010)

[Gene Ontology (GO) pathway enrichment analysis 8](#_Toc215991011)

[Gene set variation analysis (GSVA) 8](#_Toc215991012)

[Pathway score estimation 9](#_Toc215991013)

[Single-cell regulatory network inference and clustering (SCENIC) analysis 9](#_Toc215991014)

[Cell-cell communication analysis 9](#_Toc215991015)

[Pseudotime trajectory analysis 10](#_Toc215991016)

[Supplementary Figure Legends 10](#_Toc215991017)

[Supplementary Reference 16](#_Toc215991018)

# Supplementary Methods

### Sample collection details

Patients in both groups received identical standardized post-transplant immunosuppressive regimens. Briefly, basiliximab (20mg) was administered intravenously during the surgery and again on POD4. Beginning on POD4, patients received combination therapy with tacrolimus and mycophenolic acid or sirolimus. The initial tacrolimus dose was 0.04 mg/kg/d, with target trough concentrations of 8-10 ng/ml during the first three months, consistent with our previously published clinical trial[1]. Sample collection procedures followed the protocols established in our previous study[2]. Briefly, blood samples were collected in 10 ml anticoagulation tubes containing EDTA (BD Biosciences, Cat. no. BD-366643, USA). Liver tissues were washed in normal saline (4°C) to remove blood and secretions, and then placed immediately in a D10 resuspension buffer, which contained culture medium (DMEM medium; Gibco™, Cat. no. 11965092, USA) with 10% fetal bovine serum (FBS; Gibco™, Cat. no. 10099141, USA). Liver biopsies were kept on ice and delivered to the laboratory within 40 mins for further processing. Other samples for immunohistochemistry assays, immunofluorescence staining, and cytokine analysis were collected from additional donors at the FAH-SYSU.

### Immunostaining assays

For immunohistochemistry (IHC) analysis, tissue sections were stained with secondary antibodies for 1 h at room temperature, and then DAB chromogenic immunoprecipitation was performed. All sections were processed with hematoxylin for counterstaining. The antibodies of IHC used in this study are listed in the **Table. S5**. IHC evaluation was based on the staining intensity and percentage of stained cells, which was independently completed by two pathologists at FAH-SYSU.

For immunofluorescence assays, after incubation with primary antibodies, tissue sections were stained with multiple secondary antibodies at room temperature. Slides were counterstained with DAPI (D9542; Sigma) at 2 μg/mL in phosphate-buffered saline (PBS) (21-040-CVC; Corning) for 2 min, mounted with fluorescence mounting medium (ZLI-9556; ZSGB-BIO) and stored at 4°C. The slides were imaged using a fluorescence microscope (Nikon Eclipse Ni-E; Nikon, Japan). The antibodies of immunofluorescence are listed in the **Table. S5**. Secondary antibodies included goat anti-mouse IgG (H+L) (ZF-0512; ZSGB-BIO) and goat anti-Rabbit IgG (H+L) (ZF-0516; ZSGB-BIO).

### Flow cytometry assays

Peripheral blood was collected from recipients 2h after LT, and peripheral blood mononuclear cells (PBMCs) were isolated using Ficoll density gradient centrifugation. The mononuclear cell layer was harvested, washed twice with PBS, and processed for flow cytometry. For surface staining, PBMCs were incubated with the fluorochrome-conjugated antibodies for 15 minutes. Cells were fixed and permeabilized using eBioscience^TM^ Intracellular Fixation & Permeabilization Buffer Set (Invitrogen, Waltham, USA) according to the manufacturer’s instructions, followed by intracellular staining with fluorochrome-conjugated antibodies for 30 minutes. The fluorochrome-conjugated antibodies used in this study are listed in the **Table. S5**. Data were acquired using BD LSRFortessaTM X-20 and analyzed by the Flowjo software (version 10.8).

### Monocytes isolation from PBMCs

To achieve high purity monocyte isolation, CD14^+^ monocytes were purified from human PBMCs collected at PR using magnetic-activated cell sorting (MACS). Briefly, PBMCs were incubated with anti-CD14 MicroBeads (Miltenyi Biotec, Bergisch-Gladbach, Germany) for 15 minutes at 4 ℃. Following two washes, the cell-bead suspension was applied onto an MS Column positioned in the magnetic field of an MS MACS Separator (Miltenyi Biotec, Bergisch-Gladbach, Germany). After three additional washes, magnetically retained CD14^+^ monocytes were recovered as the positively selected fraction. The purity of isolated CD14^+^ monocytes was greater than 96%, as confirmed by flow cytometric analysis.

### Western blot assays

Total protein lysates of CD14^+^ monocytes were extracted using RIPA buffer (Beyotime, Shanghai, China) containing protease and phosphatase inhibitors (Selleck, Houston, USA. The protein concentration was determined by Pierce^TM^ BCA protein assay kit (Invitrogen, Waltham, USA) following the manufacturer’s instruction. Protein samples were then separated by SDS-polyacrylamide gel electrophoresis (SDS-PAGE) and electrophoretically transferred onto PVDF membranes (Merck Millipore, Billerica, USA). The membranes were then blocked with 5% bovine serum albumin (BSA, Sangon Biotech, Shanghai, China) at room temperature for 1 hour. After blocking, the membranes were incubated overnight at 4 °C with the following primary antibodies: β-actin, HIF-1α, STAT3, Phospho-STAT3 (Tyr705). Following three washes with TBST, the membranes were incubated with corresponding horseradish peroxidase (HRP)-conjugated secondary antibodies (Cell Signaling Technology, Danvers, USA) for 1 hour at room temperature. Immunoreactive bands were detected using an enhanced chemiluminescence (ECL) reagent (Fdbio Science, Shanghai, China). Detailed information of all primary and secondary antibodies used in this experiment is provided in the **Table. S5**.

### Cell culture under hypoxia-mimicking condition

PBMCs were isolated from healthy donors and culture in RPMI. Cells were treated with 100 μM cobalt chloride (CoCl_2_) (Sigma-Aldrich, St. Louis, USA) for 16h at 37°C, followed by a 24h recovery period. After treatment, PBMCs were collected for flow cytometric analysis of CD14, FPR1, MHC-I, HIF-1α, STAT3, phospho-STAT3 (Tyr705), IL-6, IL-1β, TNF-α, and NLRP3. Protein expression of β-actin, HIF-1α, STAT3, and phospho-STAT3 (Tyr705) was assessed by western blotting.

### Estimation of infiltrated immune cell proportion using CIBERSORT

To investigate whether IFLT and CLT influence immune cell infiltration in liver tissues at 2h post-transplantation, we used the CIBERSORT algorithm to deconvolute the immune cell composition from bulk RNA sequencing (RNA-seq) data. The bulk RNA-seq data comprised 28 samples from each IFLT and 28 samples from CLT patients retrieved from our published dataset (GSE113024) [3]. The immune cell composition of each sample was measured using the CIBERSORT [4] R package (v1.03). As recommended, we normalized the gene expression matrices from bulk RNA-seq data using transcripts per million (TPM) as the input for CIBERSORT analysis. The “LM22.txt” expression profiles, used as references, were downloaded from the CIBERSORT website (<https://cibersort.stanford.edu/download.php>) [5]. The cell compositions were consolidated into major cell clusters, including B/plasma cells, T cells, NK cells, myeloid cells, eosinophils, and neutrophils. Boxplots show the proportion of infiltrating immune cells. Statistical significance between the IFLT and CLT groups was determined using an unpaired two-sided Wilcoxon test.

### Tissue dissociation and single-cell suspension preparations

All samples were processed within 1.5 hours following our previously established protocols [2]. Briefly, liver tissues were cut into less than 1 mm and incubated with a digestive solution cocktail, followed by neutralization with the D10 buffer. The cell suspensions were passed through a 40 µm cell strainer (BD, Cat. no. 352350, USA) and then resuspended in the D10 buffer at a concentration of 50-500 million cells per milliliter. The periphery immune cells were isolated from blood samples using density gradient centrifugation (Lymphocyte Separation Medium (Tian Jin Hao Yang Biological Manufacture Co., Ltd, Cat. no. LTS10770125, China). The resulting single-cell suspension was stained with Calcein AM (Component A: AM) and Ethidium homodimer-1 (Component B: EH) in LIVE/DEAD Viability/Cytotoxicity Kit (Invitrogen, Cat. no. L3224, USA) and CD45 antibody (BioLegend, Cat. no. 304037, USA) for 25 min on ice. Only the CD45^+^AM^+^EH^-^ cells were collected using fluorescence-activated cell sorting (FACS; BD Biosciences, FACS Aria™ III, USA) for each sample.

### Single-cell library preparation

For blood samples, two peripheral blood samples from different donors were pooled equally to form a mixture before loading to 10X Channel. The single-cell suspensions from peripheral blood mixture and liver tissues were counted using a Cellometer Auto 2000 (Nexcelom Bioscience, Cellometer Auto 2000, USA), and were resuspended to about 1,000 cells/μl in 1 x DPBS with 0.04% BSA. Based on the standard procedure of the Chromium Single Cell V(D)J kit V2, approximately 25,000 cells from peripheral blood mixture and 18,000 cells from liver tissues were loaded into a CHROMIUM channel (10X Genomics, CA, USA). In brief, mRNA transcripts were ligated with barcoded indexes at the 5’-end and reverse transcribed into cDNA using GemCode technology (10X Genomics, USA). cDNA libraries were constructed, including the full-length V(D)J segments of T cell receptor (TCR) and 5’-end fragments for gene expression. High-throughput sequencing was performed based on these libraries.

### Single-cell RNA sequencing (scRNA-seq) data processing

The 5’-end cDNA and TCR libraries were sequenced on DNBSEQ-T7 (MGI, Shenzhen, China) instruments with pared-end read length of 150 bp. cDNA reads were aligned to human reference genome (hg38) on CellRanger (“count” function, v5.0.0, 10X Genomics). And TCR reads were aligned to the human reference VDJ dataset (http://cf.10Xgenomics.com/supp/cell-vdj/refdata-cellranger-vdj-GRCh38-alts-ensembl-5.0.0.tar.gz) on CellRanger (“vdj” function, v5.0.0, 10x Genomics). “Forcecells” were set as 15,000 and other parameters as default. A gene expression matrix was generated using CellRanger in the “filter_feature_bc_matrix” file for further analyses.

### Doublet identification and cell clustering

The downstream single-cell analyses were performed using R software (v4.1.3). Doublet identification and cell clustering were performed using R package DoubletFinder” (v2.0.3) [6] and Seurat (v3.2.3) [7] with default settings unless otherwise stated.

For each sample, low-quality cells (gene number < 500, UMI < 1,000, and mitochondrial gene percentage > 0.25) were removed. For the remaining cells, “NormalizedData” function was used to normalize the gene expression count matrices followed by identifying high variable genes using “FindVariableGenes” function. Normalized data was then scaled to regress UMIs and mitochondrial genes using “ScaleData” function. Then, “RunPCA” function was used to identify the variation among cells based on top 2,000 high variable genes. Doublets were identified using “DoubletFinder” (v2.0.3), with application of the same PCs from the PCA analysis. 7% doublet formation rate was assumed for each sample in a droplet channel. Optimal pK values were determined independently for each sample based on the Mean-variance normalized bimodality coefficient (BCmvn). After the removal of the doublets, the remaining cells were merged into a combined object.

We then used Harmony algorithm [8] to correct potential batch effected across samples setting sample as variation. We used “FindCluster” function to identify major cell clusters, which were visualized cells using Manifold Approximation and Projection (UMAP).

Differentially expressed genes (DEGs) were determined using the Wilcoxon test in the “FindAllMarkers” function, with significance set at an average log_2_(fold change) of at least 0.25 and a Bonferroni-adjusted p-value lower than 0.05. Candidate markers were reviewed and used for cell cluster annotation. A similar strategy was applied to recluster major cell types, including T, NK, myeloid, and B/plasma cell types.

### Identification of cell identities in the peripheral blood mixture

To map cell identities in the peripheral blood mixture to corresponding individuals, Souporcell (v2.0) was used to cluster peripheral blood cells based on their detected single nucleotide polymorphism (SNP) information [9]. Only high-quality cells were included in the Souporcell analysis. The “clusters” parameter was set to two, and SNP data from 1000 Genomes hg38 reference were applied as known genotypes to define peripheral blood cell identities. Doublets identified by Souporcell were first removed, and peripheral blood cluster identities were further confirmed by analyzing the overlap ratio of TCR clone information between peripheral blood and liver samples at EP. Peripheral blood clusters sharing the most abundant TCR clones with specific liver samples were identified as originating from the same individual.

### Identification of cells from donors and recipients

After liver transplantation, immune cells from recipients infiltrate to donors’ livers via blood circulation. To investigate the composition of different immune cells from either the donor or recipient in the grafts at the PR phase, we used Souporcell to identify cell identities following a similar strategy as previously described [9]. For each liver sample at the PR phase, high-quality cells were clustered into two groups (Cluster 0 and 1) using Souporcell. Then, we compared overlapped TCR between PR samples (Cluster 0 and 1) and EP samples (donor cells) (**Fig. S2b**). The cell cluster sharing the most abundant TCR clones with the EP sample was identified as originating from the donors, and vice versa for cells from the recipients. The approach robustly delineated cells from distinct donors within the EP samples with 99.79% accuracy, underscoring the high efficiency of this approach **(Fig. S2c and S2d)**. With the application of the approach to EP and PR samples, cells in the PR samples were independently and effectively segmented into distinct clusters, while cells in the EP samples gathered into a group **(Fig. S2e).**

### Gene Ontology (GO) pathway enrichment analysis

GO enrichment analysis was performed using the online tools Metascape (<http://metascape.org/gp/index.html>) [10]. We selected the top 150 DEGs in each cluster with log_2_(fold change) > 0.25, adjusted *P* value < 0.05 and pct.1 > 0.15 for GO enrichment analysis. GO molecular functions, GO biological processes, hallmark gene sets, reactome gene sets, KEGG pathways, WikiPathways, and BioCarta gene sets were included to conduct gene enrichment analysis.

### Gene set variation analysis (GSVA)

GSVA was performed to estimate the pathway activity in an unsupervised manner [11]. We used R package GSVA (v1.48.2) to analyze the activity difference of target pathways based on gene expression profiles. All the analyzed pathways included in our study are listed in detail in **Table. S6**. Boxplots were used to show the GSVA score of each pathway between different groups. Statistical significance between the IFLT and CLT groups was determined using the unpaired two-sided Wilcoxon test.

### Pathway score estimation

To evaluate pathway scores in individual cells, we applied “AddModuleScore” function implemented in Seurat package. The genes implemented in Reactome gene sets (REACTOME_CLASS_I_MHC_MEDIATED_ANTIGEN_PROCESSING_PRESENTATION.gmt) and metabolic gene sets (h.all.v7.0.symbols.gmt) [12] were used to calculate inflammasome complex assembly score and metabolic pathway scores. “Corrplot” package (ref) (v0.84) was used for calculating Pearson correlation coefficient between metabolic pathway and inflammatory pathways.

### Single-cell regulatory network inference and clustering (SCENIC) analysis

SCENIC (v1.1.3) is a computational software to infer the gene regulatory networks [13]. We performed the SCENIC analysis using the default parameters. The transcription factor-searching regions were restrained to 10 k kilo base from the center of the transcription start site (TSS) or 500 bp upstream of the TSSs. “RcisTarget” package (v1.6.0) and “GENIE3” package (v1.8.0) were used to identify the transcription factor binding motifs overrepresented on a gene list and network inferring, respectively. No more than 500 cells were randomly selected for each cluster (CLT vs. IFLT, 2h post-transplantation) in each cell cluster. The top 10 transcription factors, based on a decrease in fold change (CLT vs. IFLT, 2h post-transplantation), were determined to be group-specific in each cluster using an unpaired two-sided Wilcoxon test and Bonferroni’s adjustment.

### Cell-cell communication analysis

To explore cell communication between different cell types, we utilized CellPhoneDB (v2.0) [14]. Considering the large amount of overall data, 3,000 cells were randomly selected from each group. Normalized counts from the Seurat object were used to create CellPhoneDB object, and the recommended preprocessing functions were applied with default parameters. Dot plots are used to show the estimated ligand-receptor results between CLT and IFLT groups.

### Pseudotime trajectory analysis

To assess the developmental trajectory of CD8^+^ T cells, we conducted developmental trajectory analysis using Mmonocle3 (v1.0.0) [15]. Raw counts data from the Seurat object were used to create monocle3 object. “preprocess_cds” function and “align_cds” function were used to preprocess Seurat data with num_dim = 10. “reduce_dimension” function was used to visualize data. The “learn_graph” function was used to infer the path of cell development. Dim plot was used to show the developmental trajectory of CD8^+^ T cells.

# Supplementary Figure Legends


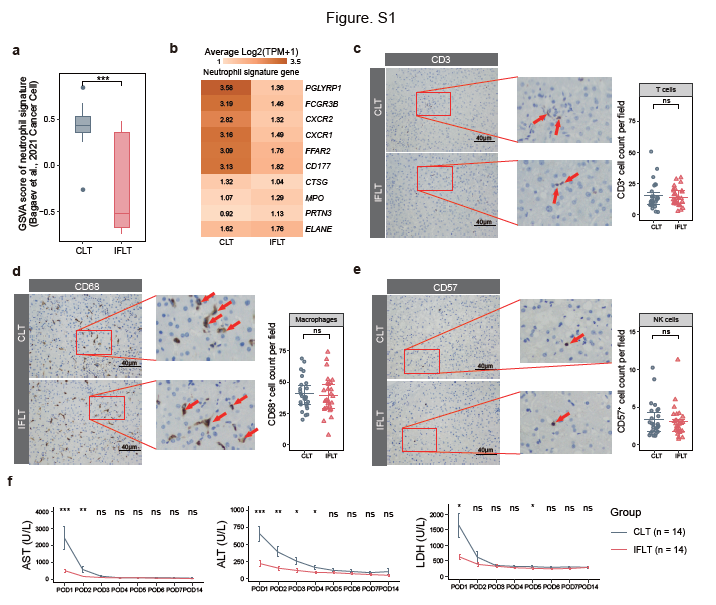


**Fig. S1. Immunostaining assay for CD3, CD68, and CD57 in liver samples, related to Fig. 1.** (a) Boxplot shows the GSVA score of neutrophil signatures (Bagaev et al., 2021 Cancer Cell) in samples based on bulk RNA-seq data between CLT (n = 14) and IFLT (n = 14) grafts. (b) Heatmap shows the average expression of neutrophil signature genes in bulk RNA-seq across CLT and IFLT patients. (c - e) Immunochemistry assays show T cells (CD3) (c), macrophages (CD68) (d), and NK cells (CD57) (e) infiltration across CLT (n = 27) and IFLT (n = 27) grafts. (f) Line plot shows the liver enzyme levels across CLT and IFLT patients over the post-operative 14 days. Ns, no significance; *, *p* < 0.05; **, *p* < 0.01; ***, *p* < 0.001; ****, *p* < 0.0001.


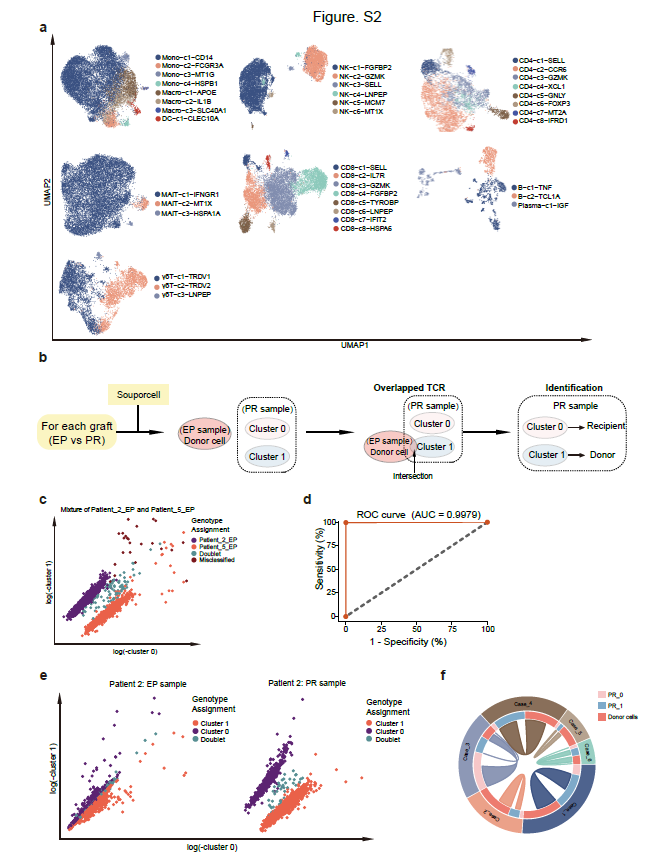


**Fig. S2. Identification of cells from donors and recipients in PR samples, related to Fig. 2.** (a) UMAP plots show the subclusters in each major cluster. (b) Schematics show identification of cells from donors or recipients in PR samples using Souporcell and TCR information. (c) Scatter plot shows the cells identified by Souporcell in manually mixed EP samples from patients 2 and 5, colored by cell origin. (d) The ROC curve shows the sensitivity and one minus specificity of cells identified by Souporcell in Fig. S2c. (e) Scatter plots show cell clusters identified by Souporcell for EP (left panel) and PR (right panel) samples. (f) Circular plot with an inner section shows cells in EP sample sharing the same TCR clone with cell clusters (Cluster 0 and Cluster 1) in the PR sample, as indicated in the outer circle. The cells sharing the same TCR clone type are lined with a line. ROC, receiver operating characteristic; EP, at the end of preservation; PR, 2 h post-reperfusion.


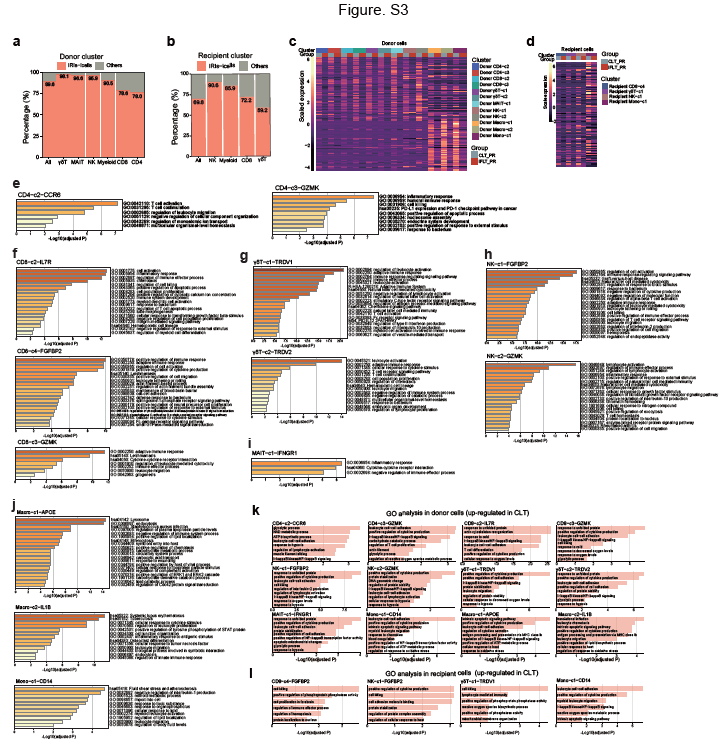


**Fig. S3. DEGs and GO enrichment analyses for donor- and recipient-derived IRIs-icells.** (a-b) Bar plot shows the percentage of donor- (a) and recipient-derived (b) IRIs-icells. (c-d) Heatmaps show average expression of selected genes across each donor- (c) and recipient-derived (d) IRIs-icells in both CLT and IFLT patients. (e-j) Bar plots showing the GO enrichment analysis based on DEGs (log_2_(fold change) > 0.25, adjusted P value < 0.05, pct.1 > 0.15) for each IRIs-icell subset: CD4^+^ T (e), CD8^+^ T (f), γδ T (g), NK (h), MAIT (i), monocyte/macrophages (j). (k-l) Bar plot shows the GO analysis pathways in donor- (k) and recipient-derived (l) IRIs-icells based on DEGs identified in C, which are up-regulated in CLT groups.


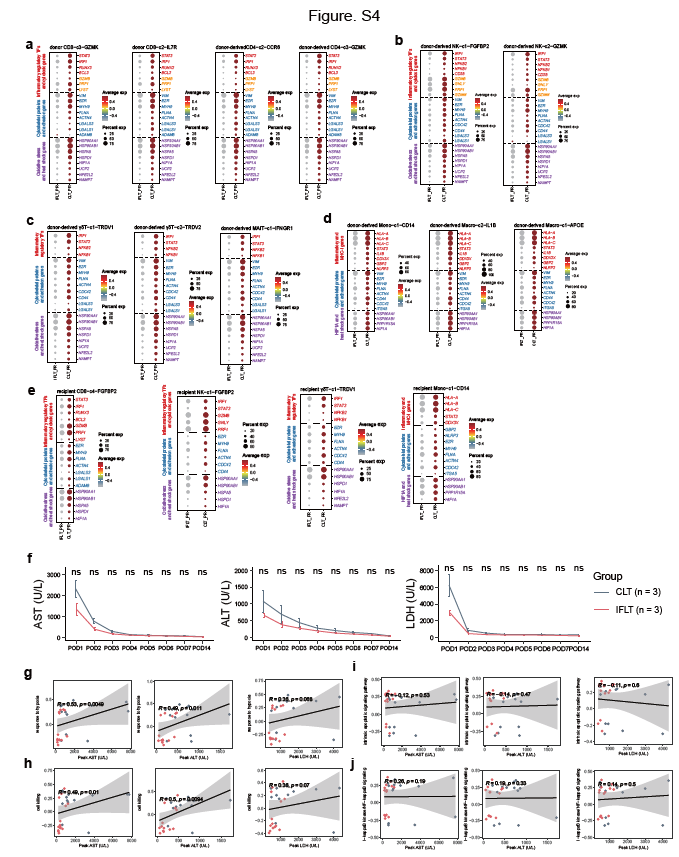


**Fig. S4. Dot plot showing the expression levels of selected DEGs in donor-derived IRIs-icells between two groups.** (a-e). Dot plots show the average expression levels of selected DEGs in donor-derived CD8^+^/CD4^+^ T cells (a), NK cells (b), γδ T/MAIT (c), monocyte/macrophages (d), and recipient-derived cells (e). (f) Line plots showing liver enzyme levels (AST, ALT, LDH) of patients included in the scRNA-seq cohort across postoperative timepoints. (g-j) Scatter plot shows the Pearson correlation between the “response to hypoxia pathway” (g), “cell killing pathway” (h), “intrinsic apoptotic signaling pathway” (i), and “I-kappaB kinase/NF-kappaB signaling pathway” (j) with peak liver enzyme levels (AST, ALT, and LDH).


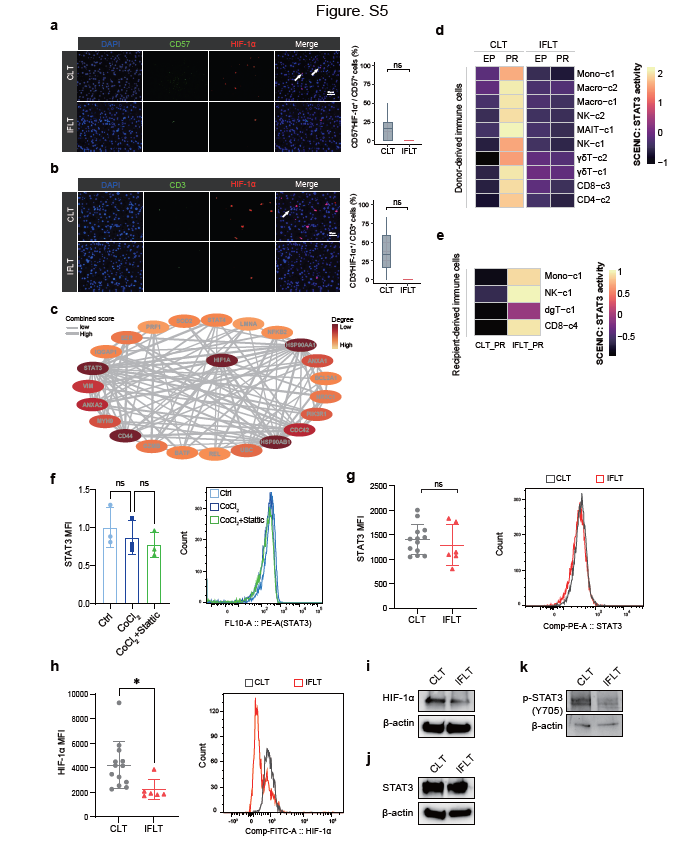


**Fig. S5. Validation of HIF1A in grafts using immunofluorescence and STAT3 activation analysis.** (a-b) Multiplex immunofluorescence staining shows expression of CD57 (green) (a) and CD3 (green) (b) with HIF-α (red) in samples collected at the PR phase across CLT and IFLT patients (CD57, n = 5 pre group; CD3, n = 3 in CLT, n = 4 in IFLT). White arrows indicate the positive cells with co-expression of CD57/HIF-α and CD3/HIF-α. Scale bars, 50 μm. (c) Network plot shows the STRING analysis results of protein-protein interaction between *HIFA* and other genes from DEGs in 12 IRIs-icells between CLT and IFLT groups. The line thickness indicated combined score (confidence score) between two genes, which the color intensity represents the degree value (the darker the color, the more interactions that gene had with other genes). (d-e) Heatmaps show transcription factor activity of STAT3 in donor- (d) and recipient-derived (e) IRIs-icells. (f) Bar plots showing the expression of STAT in monocytes under control condition, CoCl_2_ treatment, or CoCl_2_ plus the STAT3 inhibitor Stattic, measured by flow cytometry (n = 3 per group). Representative histogram plots are shown to the right of scatter plot. Monocytes were isolated from peripheral blood of healthy donors. (g-h). Scatter plots showing the Median Fluorescence Intensity (MFI) of STAT3 (g) and HIF-1α (h) measured by flow cytometry in monocytes from CLT (n = 13) and IFLT patients (n = 6) PBMC at the PR timepoint. Representative histogram plots are shown to the right of each scatter plot. (i-k) Representative Western blot analysis showing the HIF-1α (i), STAT3 (j), and p-STAT3 (k) expression in monocytes isolated from PBMCs of CLT and IFLT patients at PR. Ns, no significance; *, *p* < 0.05; **, *p* < 0.01.


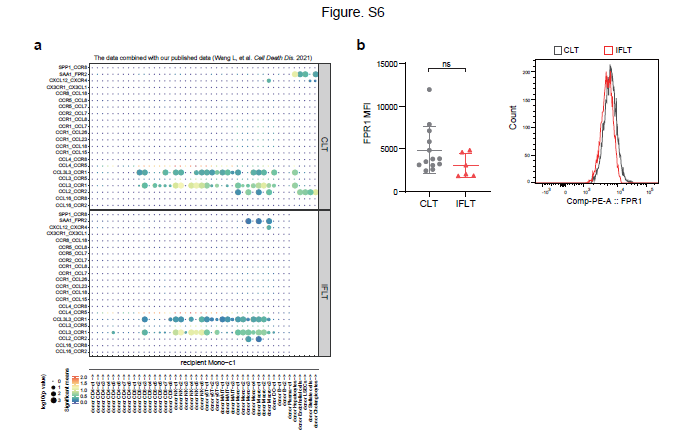


**Fig. S6. cellular interaction analysis between monocyte and other cell types (including immune cells, endothelial cells, hepatocytes, and hepatic stellate cells).** (a) Dot plot shows the interaction pairs of other chemokine signaling axes between donor-derived cells and recipient-derived Mono-c1. (b). Scatter plots showing the Median Fluorescence Intensity (MFI) of FPR1 measured by flow cytometry in monocytes from CLT (n = 13) and IFLT patients (n = 6) PBMC at the PR timepoint. Representative histogram plots are shown to the right of each scatter plot. Ns, no significance. *, *p* < 0.05.


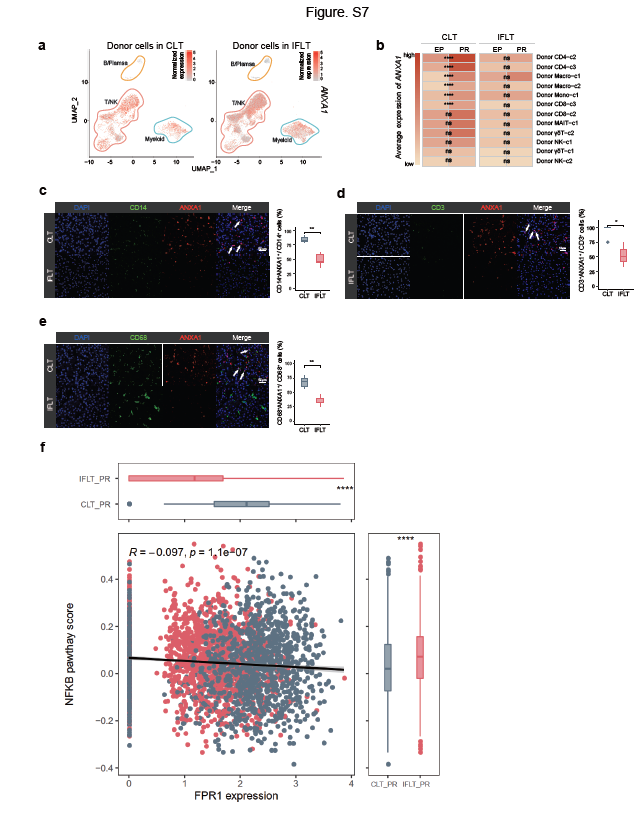


**Fig. S7. Validation of ANXA1 in multiple cell types in grafts using immunofluorescence.** (a) UMAP plots show the expression level of *ANXA1* in donor-derived cells between CLT and IFLT patients. (b) Heatmap shows the average expression level of *ANXA1* in different IRIs-icells clusters between EP and PR samples across CLT and IFLT groups. Log_2_(fold change) (PR vs EP) greater than 1 is considered significant. (c-e) Multiplex immunofluorescence staining shows expression of CD14 (green) (c), CD3 (green) (d), and CD68 (green) (e) with ANXA1 (red) in PR samples between CLT and IFLT patients (CD14, CD68: n = 5 pre group; CD3: n = 5 in CLT, n = 2 in IFLT). White arrow indicates the positive cells with co-expression of CD68/CD14/CD3 and ANXA1. Scale bars, 50 μm. (f) Scatter plot shows the Pearson correlation between *FPR1* and *RELA* expression levels in recipient-derived Mono-c1. Ns, no significance; *, *p* < 0.05; **, *p* < 0.01; ***, *p* < 0.001; ****, *p* < 0.0001.


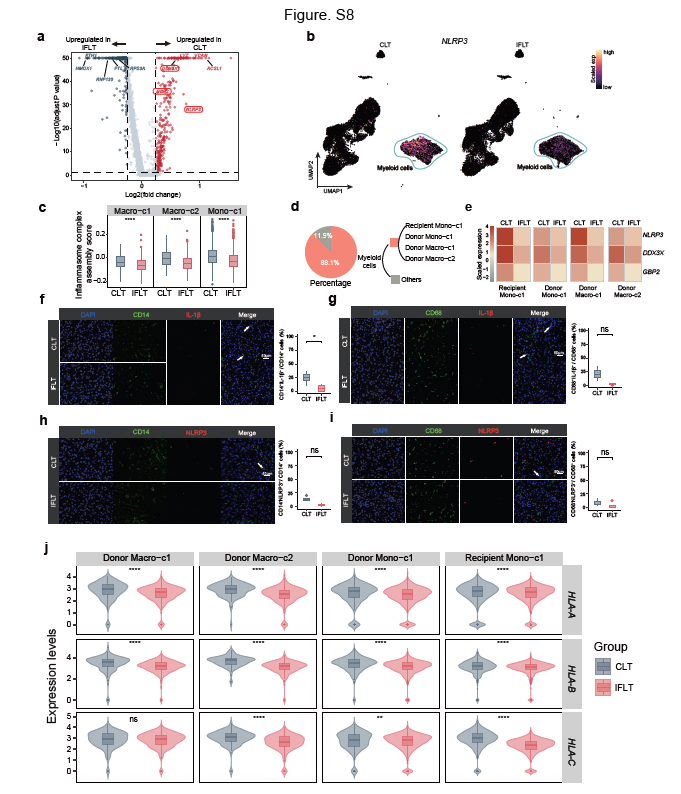


**Fig. S8. Inflammatory features in monocytes and macrophages across CLT and IFLT grafts.** (a) Volcano plot shows DEGs in monocytes and macrophages (CLT vs IFLT). (b) UMAP plots show the expression level of *NLRP3* in CLT and IFLT grafts. (c) Boxplots show the module score of inflammasome pathway across CLT and IFLT grafts in monocytes and macrophages. (d) Circular bar plot shows the percentage of donor-derived Macro-c1, Macro-c2, and Mono-c1, and recipient-derived Mono-c1 in myeloid cells. (e) Heatmap shows the scaled expression of *NLRP3*, *DDX3X*, and *GBP2* in donor-derived Macro-c1, Macro-c2, and Mono-c1, and recipient-derived Mono-c1. (f-g) Multi-colour immunofluorescence in CLT and IFLT grafts shows the expression of CD14 (green) and CD68 (green) with IL-1β (red) in samples collected at the PR phase across CLT and IFLT patients (CD14: n = 5 in CLT, n = 4 in IFLT; CD68: n = 3 in CLT, n = 4 in IFLT). (h-i) Multi-colour immunofluorescence in CLT and IFLT grafts shows the expression of CD14 (green) and CD68 (green) with NLRP3 (red) in samples collected at the PR phase across CLT and IFLT patients (CD14: n = 4 in CLT, n = 2 in IFLT; CD68: n = 5 in CLT, n = 5 in IFLT). (j) Violin plots show the expression levels of MHC-I molecular genes in donor- and recipient-derived monocytes and macrophages between CLT and IFLT grafts. Ns, no significance; *, *p* < 0.05; **, *p* < 0.01; ***, *p* < 0.001; ****, *p* < 0.0001.


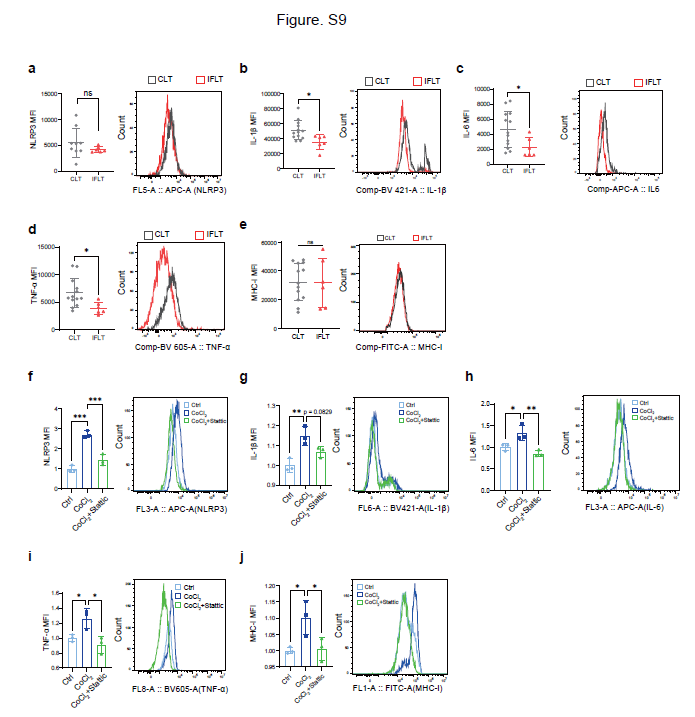


**Fig. S9. MHC-I molecule expression in monocytes and macrophages across CLT and IFLT grafts.** (a-e) Scatter plots showing the MFI of NLRP3 (a), IL-1β (b), IL-6 (c), TNF-α (d), and MHC-I (e) in monocytes from CLT (n =9 for NLRP3, n = 13 for IL-1β/IL-6/TNF-α/MHC-I) and IFLT patients (n = 6) PBMC at the PR timepoint. Representative histogram plots are shown to the right of each scatter plot. (f-j) Bar plots showing the expression of NLRP3 (f), IL-1β (g), IL-6 (h), TNF-α (i), and MHC-I (j) in monocytes under control condition, CoCl_2_ treatment, or CoCl_2_ plus the STAT3 inhibitor Stattic, measured by flow cytometry (n = 3 per group). Representative histogram plots are shown to the right of each scatter plot. Monocytes used in f-j were isolated from peripheral blood of healthy individuals. Ns, no significance; *, *p* < 0.05; **, *p* < 0.01; ***, *p* < 0.001; ****, *p* < 0.0001.


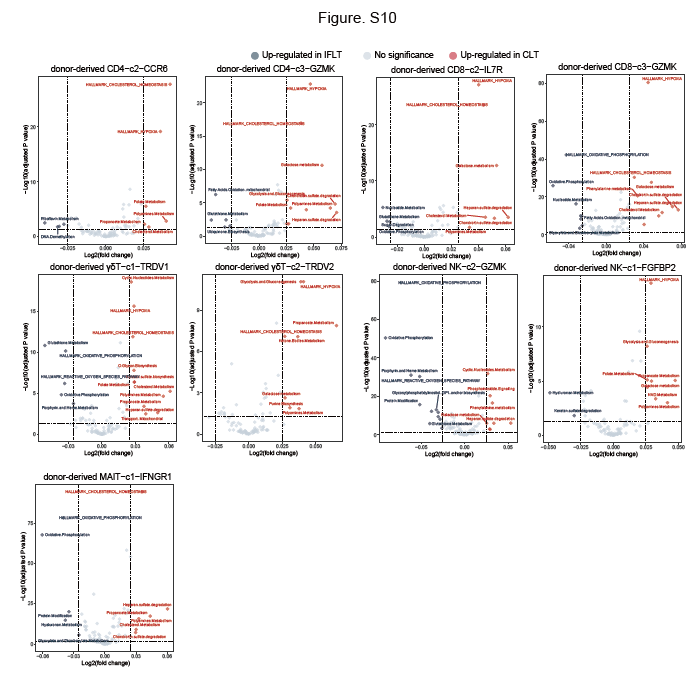


**Fig. S10. Different metabolic pathways analysis of in donor-derived IRIs-icell clusters.** Volcano plot shows differentially expressed metabolic pathways in the remaining donor-derived IRIs-icell clusters in PR samples (CLT vs IFLT).


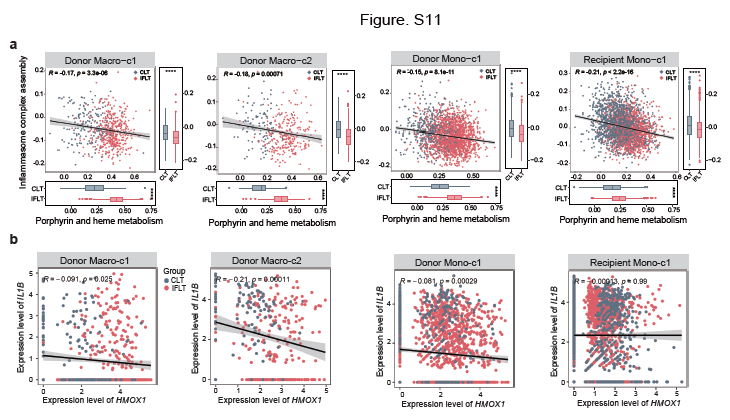


**Fig. S11. Correlation betwee metabolism and inflammsome pathway and related genes in monocytes and macrophages.** (a). Scatter plots show the Pearson correlation between the module score of “inflammasome complex assembly” and “Porphyrin and Heme Metabolism” in donor-derived Macro-c1, Macro-c, and Mono-c1, and recipient-derived Mono-c1 between CLT and IFLT patients. (b) Scatter plots show the Pearson correlation between expression levels of *HMOX1* and *IL1B* in donor-derived Macro-c1, Macro-c, and Mono-c1, and recipient-derived Mono-c1 between CLT and IFLT patients.


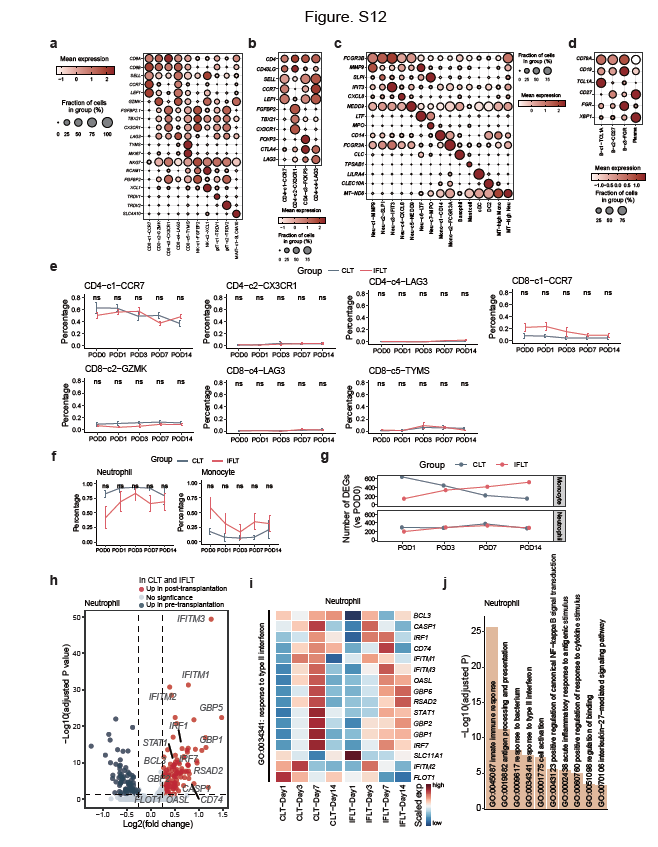


**Fig. S12. Basic information of cell clusters, related to Fig. 6.** (a-d) Dot plots show normalized expression levels of marker genes in T cells (a-b), myeloid cells (c), and B/plasma cells (d). (e) Line plots show the cell proportions of the remaining cell clusters (mean ± SEM) in T cells. (f) Line plots show the changes in percentage of neutrophils and monocytes across CLT and IFLT recipients in different time points. (g) Line plots show the number of DEGs (the time points after transplantation *vs* POD 0) between CLT and IFLT recipients in neutrophils and monocytes. (h) Volcano plot shows DEGs (post-transplant *vs* pre-transplant) in neutrophils. (i) Heatmap plot shows average expression levels of “GO:0034341 response to type II interferon” genes in neutrophils. (j) Bar plot shows the top 11 enriched GO pathways in post-transplantation. Ns, no significance.


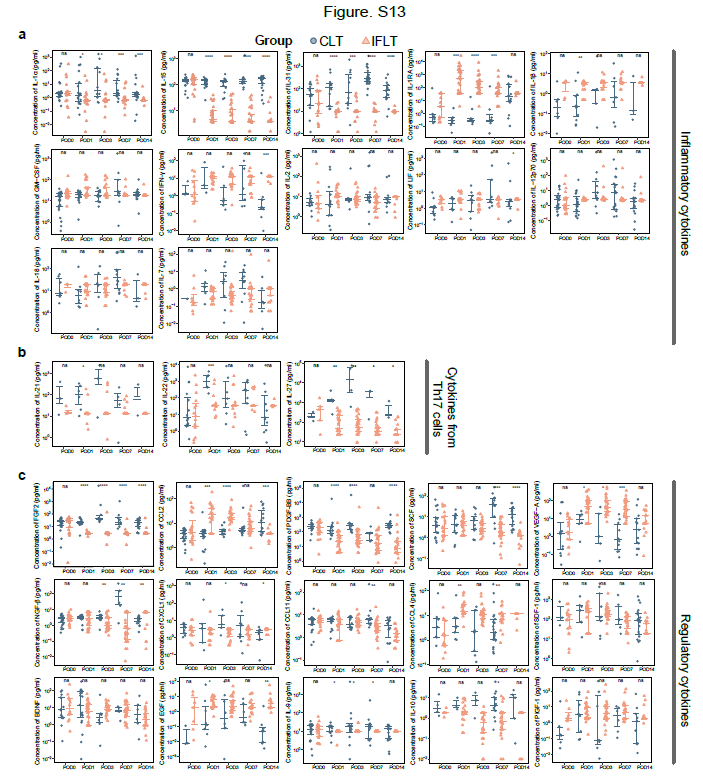


**Fig. S13. Cytokine analysis in peripheral blood across CLT and IFLT recipients.** (a) Jitter plots show the concentration of inflammatory cytokines in postoperative serum at different time points across CLT and IFLT recipients (n = 30), including IL-1α, IL-15, IL-31, IL-1RA, IL-1β, GM-CSF, IFN-γ, IL-2, LIF, IL-12p70, IL-18, and IL-7. (b) Jitter plots show the concentration of cytokines from Th17 in postoperative serum at different time points across CLT and IFLT recipients (n = 30), including IL-21, IL-22, and IL-27. Unpaired two-sided Wilcoxon test. (c) Jitter plots show the concentration of regulatory cytokines in postoperative serum at different time points across CLT and IFLT recipients (n = 30), including FGF2, CCL2, PDGF-BB, SCF, VEGF-A, NGF-β, CXCL1, CCL11, CCL4, SDF-1, BDNF, EGF, IL-9, IL-10, and PIGF-1. Ns, no significance; *, *p* < 0.05; **, *p* < 0.01; ***, *p* < 0.001; ****, *p* < 0.0001.

# Supplementary Reference

[1] Z. Guo, Q. Zhao, Z. Jia, C. Huang, D. Wang, W. Ju, J. Zhang, L. Yang, S. Huang, M. Chen, X. Zhu, A. Hu, Y. Ma, L. Wu, Y. Chen, M. Han, Y. Tang, G. Wang, L. Wang, L. Li, W. Xiong, Z. Zhang, Y. Shen, Z. Tang, C. Zhu, X. Chen, X. Hu, Y. Guo, H. Chen, Y. Ma, T. Zhang, S. Huang, P. Zeng, S. Lai, T. Wang, Z. Chen, J. Gong, J. Yu, C. Sun, C. Li, H. Tan, Y. Liu, Y. Dong, C. Sun, B. Liao, J. Ren, Z. Zhou, S. Andrea, N. Björn, C. Cai, F. Gong, J. Rong, W. Huang, X. Guan, P.-A. Clavien, T. G. Stefan, J. Huang, X. He, *J Hepatol* **2023**, *79* (2), 394, <https://doi.org/10.1016/j.jhep.2023.04.010>.

[2] S. He, L. H. Wang, Y. Liu, Y. Q. Li, H. T. Chen, J. H. Xu, W. Peng, G. W. Lin, P. P. Wei, B. Li, X. Xia, D. Wang, J. X. Bei, X. He, Z. Guo, *Genome Biol* **2020**, *21* (1), 294, <https://doi.org/10.1186/s13059-020-02210-0>.

[3] Z. Guo, J. Xu, S. Huang, M. Yin, Q. Zhao, W. Ju, D. Wang, N. Gao, C. Huang, L. Yang, M. Chen, Z. Zhang, Z. Zhu, L. Wang, C. Zhu, Y. Zhang, Y. Tang, H. Chen, K. Liu, Y. Lu, Y. Ma, A. Hu, Y. Chen, X. Zhu, X. He, *Clin Transl Med* **2022**, *12* (4), e546, <https://doi.org/10.1002/ctm2.546>.

[4] A. M. Newman, C. L. Liu, M. R. Green, A. J. Gentles, W. Feng, Y. Xu, C. D. Hoang, M. Diehn, A. A. Alizadeh, *Nat Methods* **2015**, *12* (5), 453, <https://doi.org/10.1038/nmeth.3337>.

[5] B. Chen, M. S. Khodadoust, C. L. Liu, A. M. Newman, A. A. Alizadeh, *Methods In Molecular Biology (Clifton, N.J.)* **2018**, *1711*, 243, <https://doi.org/10.1007/978-1-4939-7493-1_12>.

[6] C. S. McGinnis, L. M. Murrow, Z. J. Gartner, *Cell Systems* **2019**, *8* (4), <https://doi.org/10.1016/j.cels.2019.03.003>.

[7] T. Stuart, A. Butler, P. Hoffman, C. Hafemeister, E. Papalexi, W. M. Mauck, Y. Hao, M. Stoeckius, P. Smibert, R. Satija, *Cell* **2019**, *177* (7), <https://doi.org/10.1016/j.cell.2019.05.031>.

[8] I. Korsunsky, N. Millard, J. Fan, K. Slowikowski, F. Zhang, K. Wei, Y. Baglaenko, M. Brenner, P.-r. Loh, S. Raychaudhuri, *Nat Methods* **2019**, *16* (12), 1289, <https://doi.org/10.1038/s41592-019-0619-0>.

[9] H. Heaton, A. M. Talman, A. Knights, M. Imaz, D. J. Gaffney, R. Durbin, M. Hemberg, M. K. N. Lawniczak, *Nat Methods* **2020**, *17* (6), 615, <https://doi.org/10.1038/s41592-020-0820-1>.

[10] Y. Zhou, B. Zhou, L. Pache, M. Chang, A. H. Khodabakhshi, O. Tanaseichuk, C. Benner, S. K. Chanda, *Nat Commun* **2019**, *10* (1), 1523, <https://doi.org/10.1038/s41467-019-09234-6>.

[11] S. Hänzelmann, R. Castelo, J. Guinney, *BMC Bioinformatics* **2013**, *14*, 7, <https://doi.org/10.1186/1471-2105-14-7>.

[12] E. Gaude, C. Frezza, *Nat Commun* **2016**, *7*, 13041, <https://doi.org/10.1038/ncomms13041>.

[13] S. Aibar, C. B. González-Blas, T. Moerman, V. A. Huynh-Thu, H. Imrichova, G. Hulselmans, F. Rambow, J.-C. Marine, P. Geurts, J. Aerts, J. van den Oord, Z. K. Atak, J. Wouters, S. Aerts, *Nat Methods* **2017**, *14* (11), 1083, <https://doi.org/10.1038/nmeth.4463>.

[14] R. Vento-Tormo, M. Efremova, R. A. Botting, M. Y. Turco, M. Vento-Tormo, K. B. Meyer, J. E. Park, E. Stephenson, K. Polański, A. Goncalves, L. Gardner, S. Holmqvist, J. Henriksson, A. Zou, A. M. Sharkey, B. Millar, B. Innes, L. Wood, A. Wilbrey-Clark, R. P. Payne, M. A. Ivarsson, S. Lisgo, A. Filby, D. H. Rowitch, J. N. Bulmer, G. J. Wright, M. J. T. Stubbington, M. Haniffa, A. Moffett, S. A. Teichmann, *Nature* **2018**, *563* (7731), 347, <https://doi.org/10.1038/s41586-018-0698-6>.

[15] J. Cao, M. Spielmann, X. Qiu, X. Huang, D. M. Ibrahim, A. J. Hill, F. Zhang, S. Mundlos, L. Christiansen, F. J. Steemers, C. Trapnell, J. Shendure, *Nature* **2019**, *566* (7745), 496, <https://doi.org/10.1038/s41586-019-0969-x>.
